# Hydrogel-Mediated Sustained Delivery of Corneal Epithelial Extracellular Vesicles: A Strategy for Enhanced Corneal Regeneration

**DOI:** 10.1021/acsomega.5c01135

**Published:** 2025-08-14

**Authors:** Jenny Rosenquist Lybecker, Ann Van de Ven, Ken Braesch-Andersen, David Juriga, Norein Norein, Per Hansson, Ayan Samanta

**Affiliations:** † Macromolecular Chemistry, Department of Chemistry–Ångström Laboratory, 8097 Uppsala University, Box 538, 751 21 Uppsala, Sweden; ‡ Thoracic Surgery, Department of Surgical Sciences, Uppsala University, 751 85 Uppsala, Sweden; § Pharmaceutical Physical Chemistry, Department of Medicinal Chemistry, Uppsala University, Box 574, 751 23 Uppsala, Sweden

## Abstract

Extracellular vesicles (EVs) derived from corneal epithelial cells have shown great promise in promoting corneal wound healing and stromal regeneration, but they face challenges with rapid clearance from the eye. This study addresses these challenges by developing a biocompatible collagen-hydrogel sustained delivery system. We successfully isolated, purified, and characterized corneal epithelial EVs (CE-EVs), assessed their efficacy in corneal epithelial healing in vitro, and demonstrated their sustained delivery over 10 days followed by an on-demand release through enzymatic degradation of the hydrogel, which mimics the in vivo scenario. To develop a microscale understanding of the EV diffusion inside the hydrogel matrix, we probed the hydrogel network with several model compounds and nanoparticles by using advanced confocal microscopy analyses, followed by fitting our results to established diffusion models. Our findings suggest this innovative approach offers a safe and effective strategy to promote corneal wound healing. This technology has the potential to revolutionize corneal injury treatment and improve patient outcomes. Moreover, the possibility to tailor EV-release kinetics broadens the scope of EV research in clinical practices, as varying short- and long-term release profiles will be required for diverse medical applications.

## Graphical abstract

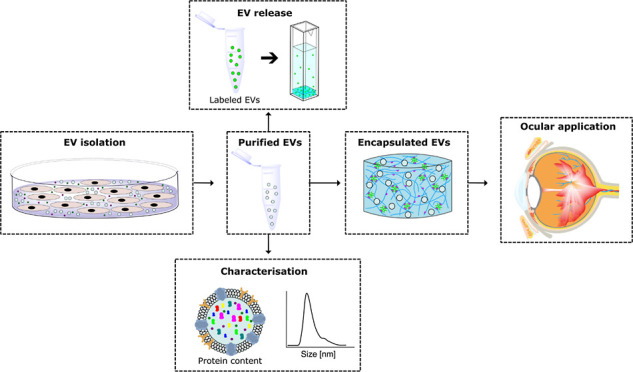

## 1 Introduction

The cornea is the transparent outermost layer of the eye and its primary functions are to serve as both a physical barrier for the interior of the eye and contribute to the refraction and focusing of light onto the lens and retina.^1^ The cornea comprises five distinct layers: the epithelium, Bowman’s layer, stroma, Descemet’s membrane, and endothelium. The structural integrity and the intricate cellular communication within and between these layers are critical for maintaining the transparency and functionality of the corneal tissue.^2^ Corneal injury may arise from chemical or thermal burns, mechanical trauma, infections, or underlying pathological conditions.^3^ Minor corneal abrasions can typically heal by itself within a couple of days.^4^ On the contrary, in the event of more severe injuries, such as corneal lacerations and perforations, the cornea’s transparency may be compromised which can lead to corneal blindness.^2^ Corneal perforation is an emergent condition requiring prompt treatment to seal the eye and restore the eye’s anatomy.^5^ The treatments range from corneal gluing to corneal transplantation by donor corneas. For small perforations (up to ∼2–3 mm), gluing is the most common treatment and there are two major variants; cyanoacrylate tissue adhesive (CTA, nonbiologic) and fibrin glue (biologic).^6,7^ CTA is the stronger one of the two but the drawbacks include tissue toxicity due to an unreacted monomer and release of formaldehyde due to degradation,^8^ rapid polymerization^6^ leading to a very short time window for application during surgery, highly exothermic reaction, nonbiodegradability, foreign-body reactions, and inconsistent adhesion on wet surfaces.^6^ Further, CTA often induces neovascularization which also can lead to corneal blindness.^9^ Fibrin glue on the other hand is nontoxic and completely biodegradable. However, as it originates from donor blood, there is a risk of transmitting viral or prion diseases.^6,10^ It has also been shown that fibrin glue inhibits corneal epithelialization, delaying the healing process.^11^ An adhesive possessing the biocompatibility of fibrin glue without impeding epithelialization would therefore be highly advantageous. In the event of larger perforations (>2–3 mm), keratoplasty becomes necessary.^6^ However, due to donor shortages, a considerable number of patients are unable to access this treatment^12^ emphasizing the crucial need for innovative methods.

The process of corneal wound closure is a complex and not entirely understood phenomenon, which includes apoptosis, cell migration, proliferation, differentiation, and restructuring of the extracellular matrix.^13^ Communication between the epithelium and the stroma is of great importance for these events to proceed in the correct way and this communication involves both the secretion of soluble proteins^14–16^ and the release of extracellular vesicles (EVs) carrying specific signaling molecules.^14–16^ EVs are lipid bilayer structures released by virtually all cell types, serving as important mediators in intercellular communication. The cargo can be a diverse array of macromolecules, including proteins, RNA, and DNA, with their composition being intricately regulated by the cellular source and for the intended recipient.^17,18^ EVs have been proposed as potential therapeutic agents in various corneal diseases such as glaucoma, dry eye disease, and corneal scarring.^3,19,20^

In our earlier work on cell-free implants in pigs,^21^ we observed that EVs were released from the regenerating epithelium. These observations led us to believe that these EVs play a role in corneal epithelial wound healing and stromal regeneration. This is supported by other studies which demonstrated that mouse corneal epithelial cell-derived EVs mediate the cell–cell communication between corneal epithelial cells, keratocytes, and endothelial cells, indicating the potential role of corneal epithelial EVs (CE-EVs) in corneal regeneration.^15^ They also demonstrated that these EVs fuse with keratocytes in vitro to induce myofibroblast differentiation,^15^ which plays a crucial role in the process of wound closure. This was further studied by McKay et al. who demonstrated that EVs originating from immortalized human corneal epithelial cells promote the differentiation of fibroblasts into myofibroblasts.^22^ More recently, Desjardins et al. isolated EVs from primary corneal epithelial cells, corneal endothelial cells, and corneal fibroblast and demonstrated that the process of corneal epithelial wound healing was accelerated by the addition of all types of corneal EVs, but particularly by corneal epithelial EVs (CE-EVs).^23^ Nonetheless, to achieve complete healing, often multiple doses of EVs were needed, which emphasizes one major drawback associated with administrating EVs. When administered alone, they exhibit a short half-life and are rapidly cleared from the body.^19^ For administration in the eye, the tear film is replaced every 5 min under normal conditions, and in the case of topical application of drugs, the tear secretion is increased.^24^ Hence, the concern is that EVs will be quickly washed away through the nasolacrimal duct leading to low bioavailability.^25^ To address this issue, it has been proposed to utilize delivery scaffolds from which EVs can be released sustainably.^19,26,27^ Nonetheless, so far, there are no reports of EVs being sustainably delivered to the eye. We hypothesized that a sustained release of EVs from a prefabricated corneal implant or an adhesive biodegradable corneal sealant could be of great importance for corneal regeneration in case of keratoplasty with a corneal implant and during repairing corneal perforations or in situ molding of corneal defects in a sutureless keratoplasty. We previously developed a loadable, chemically cross-linked adhesive hydrogel with adhesion strength comparable to fibrin glue, suitable for repairing corneal perforations and in situ molding of corneal defects in its as-prepared state.^28^ Additionally, when fully cured, these hydrogels hold potential as prefabricated corneal implants for use in keratoplasty. Herein, we report the isolation, purification, and comprehensive characterization of corneal epithelial EVs (CE-EVs) as well as their loading in the aforesaid hydrogel for sustained release. A fraction of the encapsulated CE-EVs could be released from the hydrogel over 10 days by diffusion through the hydrogel matrix, and the remaining can be released on demand through enzymatic degradation of the hydrogel. This represents a significant advancement in the field of corneal EV delivery. A perforation or epithelial wound healing would benefit from a moderate EV-release profile, while stromal regeneration after keratoplasty with a corneal implant would be supported by a very slow EV-release profile, occurring as the implant gets remodeled in vivo. Moreover, we have developed a microscale physical understanding of the EV movements within the hydrogel network and their subsequent release from the hydrogel. This is accomplished by initially fitting the experimental EV-release data to a mathematical model of one-dimensional diffusion to determine the empirical self-diffusion coefficient of the EVs within the hydrogel. Subsequently, the hydrogel network is probed with model compounds and nanoparticles, and the resulting experimental data are fitted to two distinct established microscale physical models of diffusion. Such a physical understanding will enable the future design of hydrogels suitable for various EV-release profiles as required in different medical applications. To the best of our knowledge, this is the first report on sustained EV delivery spanning weeks to months or even longer. Such an EV-delivery system holds tremendous potential for significantly advancing the treatments involving EVs in general but especially for repairing corneal injury.

## 2 Experimental Section

### 2.1 Materials and Methods

Immortalized human corneal epithelial cells (IM-HCEpiCs) (P10871-IM), IM-ocular epithelial cell medium (P60189), and collagen type-I cell culture surface coating kit (P8188) were purchased from Innoprot (Spain). Collagen-coated cell culture flasks (collagen type-I CELLCOAT) and a sterile syringe filter with a 0.22 μm cellulose acetate membrane were purchased from VWR (Sweden). Disposable poly­(ether sulfone) (PES) bottle top filters, Invitrogen LIVE/DEAD viability/cytotoxicity kit, and Gibco Opti-MEM I reduced serum medium were purchased from Fisher Scientific (Sweden). Micro BCA protein assay kit, Pierce BCA Protein Assay Kit, Gibco TrypLE Express Enzyme, Gibco Dulbecco’s phosphate-buffered saline (DPBS) (no magnesium, no calcium), CellTrace CFSE Cell Proliferation Kit, TBS with Tween (TBST), 20× Solution, Molecular Biology grade, Ultrapure, Blocker BSA (10%) in PBS, fetal bovine serum (FBS), and Invitrogen iBlot 2 Transfer Stacks (PVDF) were purchased from Thermo Fisher Scientific (Sweden). MACSPlex EV Kit IO (human) was purchased from Miltenyi Biotec Norden AB. 10× Tris/Glycine/SDS, 2× Laemmli Sample Buffer, 4–20% Mini-PROTEAN TGX Stain-Free Protein Gels, Clarity Western ECL substrate, Goat Anti-Rabbit IgG (H + L)-HRP Conjugate, and Goat Anti-Mouse IgG (H + L)-HRP Conjugate were purchased from Bio-Rad. Anti-CD81 antibody [M38] (ab79559), Anti-CD63 antibody [TS63]—BSA and Azide free (ab59479), Anti-Calnexin antibody—ER Marker (ab22595), and Anti-Fibronectin antibody [F14] (ab45688) were purchased from Abcam (Netherlands). Anti-Collagen Type-I Rabbit pAb, fluorescence isothiocyanate-labeled dextran (FITC-dextran, various molecular weights), Amicon ultra-15 centrifugal filter unit 10 kDa molecular weight cut off (MWCO), Amicon Ultra Centrifugal Filter 2 mL, 10 kDa MWCO, and Vivaspin 20 centrifugal concentrator MWCO 300 kDa were purchased from Merck (Sweden). Silica NPs were purchased from Micromod particle technology GmbH (Germany).

### 2.2 Cell Culture of IM-HCEpiC

IM-HCEpiC cells were cultured following the protocol of the supplier with minor modifications. In short, the cells were cultured using an ocular epithelial cell medium supplemented with 5% of FBS, 1% of epithelial cell growth supplements, and 1% of penicillin/streptomycin solution and kept in an incubator at 37 °C with 5% CO2. Subculturing was done when cells were ∼90% confluent by adding a TrypLE Express Enzyme. When all cells were detached, culture medium was added to inactivate the enzyme. Cells were collected by centrifuging at 200 RCF (Eppendorf Centrifuge 5810 R) for 5 min, and the pellet was resuspended in culture medium. The number of cells was counted by using an EVE automated cell counter (NanoEntek). Cells were seeded at a minimum density of 10,000 cells/cm^2^ in collagen-coated cell culture flasks (either purchased or coated in-house according to supplier’s instructions).

### 2.3 Isolation of EVs from IM-HCEpiC

IM-HCEpiCs were cultured in collagen-coated flasks until a large number of cells were achieved (passage 4–7) to be able to obtain a volume of 50–500 mL of collected media with EVs. Opti-MEM (0.1 mL/cm^2^) was added to the IM-HCEpiC when they reached ∼80% confluency. The cells were incubated with Opti-MEM for 48 h and then the supernatant was collected and centrifuged (Eppendorf Centrifuge 5810 R) at 700 RCF for 5 min at 11 °C. The new supernatant was collected and centrifuged at 1450–2000 RCF for 10–15 min (Eppendorf Centrifuge 5810 R). The supernatant was then filtered through a 0.22 μm syringe filter, the final volume was noted down, and a sample for NTA (1000 μL) was collected and stored in the fridge.

### 2.4 Cell Viability after EV Isolation

The ratio of live to dead cells was investigated after EV isolation by detaching the cells with TrypLE, followed by a centrifugation step at 200 RCF (Eppendorf Centrifuge 5810 R), the pellet was then resuspended in fresh medium, and the cells were counted using an EVE automated cell counter (NanoEntek).

### 2.5 Tangential Flow Filtration (TFF)

The setup for the tangential flow filtration included a Cole Parmer MasterFlex L/S Economy Drive 7554-90 pump, Cytiva (formerly Pall Lab) Minimate; TFF system reservoir kit, TFF system fitting kit, and a pressure gauge (4 bar, 60 psi) connected to a 300 K Minimate tangential flow filtration capsule with an omega membrane (Cytiva formerly Pall Lab). The water and DPBS used for TFF were autoclaved and sterile filtered through a 0.22 μm vacuum filter before use. The 300 kDa cut off membrane was first washed with water to remove storage buffer and salt particles. Then, the buffer was exchanged to DPBS before the isolated supernatant from IM-HCEpiC was run through the membrane in the TFF. First, the sample was concentrated down to between 100 and 200 mL. Then, the sample was run through at a high speed but with low pressure (the waste tubing flow was limited to ∼1–1.5 drop/s and the speed of the pump was 60–100 mL/min and pressure was kept at ∼0.5–1.5 bar). To achieve a good purity (99.9%), seven times the amount of DPBS relative to the concentrated sample was run through. The sample was then concentrated down to <50 mL and collected. The volume was noted down and the sample was filtered through a 0.22 μm filter. A sample (1000 μL) for NTA was collected and stored in the fridge.

### 2.6 Filtration through an Amicon Filter

For further purification and concentration, a 10 kDa Amicon filter was used. Fifteen mL portions of the sample were centrifuged at 1450–3000 RCF for 40 min (Eppendorf Centrifuge 5810 R). To collect the EVs, the filter was rinsed several times with the concentrate, and the bottom of the filter was scratched with a 200 μL pipet tip. Afterward, 200 μL of DPBS was added, and the procedure was repeated. The collected EVs were stored in an Eppendorf Biopur in the fridge to avoid degradation, and 10 μL was put aside for NTA measurements.

### 2.7 Nanoparticle Tracking Analysis (NTA)

NTA was performed with either a NanoSight NS500 with an NS500 G532 nm Laser Module (Malvern Panalytical) or a Nanosight NS300 with a 488 nm (Blue) laser module (Malvern Panalytical). The water and DPBS used for NTA were autoclaved and sterile filtered through a 0.22 μm vacuum filter before use. Samples were diluted in DPBS to achieve ∼50–100 particles per frame. The temperature of the experiment was set to 23.3 °C and 5 videos of 30 s were recorded and analyzed by the Nanosight software NTA version 3.2 or 3.4.

### 2.8 Surface Epitope Labeling of the EVs with the MACSPlex EV Kit

Labeling of the EVs was performed according to the MACSPlex EV kit instructions. In short, 6.43 × 10^10^ particles (EVs) were used in 120 μL total volume (diluted with MACSPlex buffer). Only MACSPlex buffer was used as the blank. The MACSPlex EV capture beads were vortexed and 15 μL was added to both the sample and the blank. The sample and blank were then incubated overnight at room temperature and protected from light using a VWR tube rotator on a permanent run (18 rpm). The next day, 500 μL of buffer was added and centrifuged at 3000 RCF (High-speed Microcentrifuge CT15RE, VWR Hitachi) for 5 min. 500 μL of the supernatant was then aspirated. Five μL of each MACSPlex EV detection reagent CD9, CD63, and CD81 was added to each tube and mixed by pipetting up and down. The samples were incubated for 1 h at room temperature protected from light using a VWR Tube Rotator on a permanent run (18 rpm). Then, 500 μL of buffer was added and the samples were centrifuged at 3000 RCF for 5 min (high-speed Microcentrifuge CT15RE, VWR Hitachi), followed by aspiration of 500 μL of the supernatant. An additional 500 μL of buffer was added and incubated for 15 min protected from light using a VWR tube rotator on a permanent run (18 rpm), followed by centrifugation at 3000 RCF for 5 min (High-speed Microcentrifuge CT15RE, VWR Hitachi) and aspiration of 500 μL of the supernatant. The sample was then measured by using FACS.

### 2.9 Fluorescence-Activated Cell Sorting (FACS)

FACS was performed on a CytoFLEX, Beckman Coulter instrument using the MACSPlex EV kit. All samples containing beads were vortexed at least 30 s before measuring. The CytoFLEX was first calibrated with MACSQuant calibration beads. Then, the blank and EV samples (bound to the MACSPlex capture beads) were measured. Events per second were kept at around 100. The gain was set to FSC 133, SSC 58, FITC 20, PE 20, PC5.5 30, PC7 100, APC 100, APC-A700 1298, APC-A750 1195, PB450 76, and KO525 47. During analysis, the signal from the blank was subtracted as the background, CD63 was set as 100%, and the other surface markers were compared to CD63.

### 2.10 Cleaning of Cells for BCA, Western Blot, and Proteomics

Cells of passage six were thawed and diluted with PBS to reach a final volume of 15 mL. It was then centrifuged at 300 RCF for 7 min (Eppendorf centrifuge 5810R) and the pellet was collected (∼35 μL) and diluted to 15 mL again in PBS. The procedure was repeated 2 more times and then the final pellet was diluted in PBS to a final volume of 135 μL.

### 2.11 Micro BCA Assay

The micro BCA assay was performed according to the supplier’s instructions with slight modifications. In short, albumin standards were prepared by dilution of the 2.0 mg/mL bovine albumin serum (BSA) with DPBS. The final concentrations were 200, 100, 50, 25, 10, 5, 2.5, and 1 μg/mL. Working reagent (WR) was prepared by mixing 25:24:1 of the Micro BCA reagent A/Micro BCA reagent B/Micro BCA reagent C. The EV sample was diluted 1:72 with DPBS to achieve a concentration in the range for the assay. Then, 150 μL of each standard and sample was pipetted into a 96-well plate, and an equal amount of WR was added. The well plate was then placed on an orbital shaker (KS 260 basic, IKA) for 30 s to mix the samples, followed by incubation at 37 °C for 2 h. The well plate was then cooled to room temperature, and the absorbance at 562 nm was measured using a Tecan Microplate Reader Spark. The absorbance of the BSA standards was then plotted against the BSA concentration to obtain a standard curve and a standard curve equation. The concentration of the EV sample could then be calculated from the standard curve equation.

### 2.12 BCA Assay

For determining the protein amount in cells and in EVs, the Pierce BCA Protein Assay Kit was used according to the manufacturer’s instructions. In short, albumin standards (2000, 1500, 1000, 750, 500, 250, 125, 25, 0 μg/mL) were prepared by dilution of the 2.0 mg/mL bovine albumin serum (BSA) with PBS. Working reagent (WR) was prepared by mixing 50 parts BCA Reagent A with 1 part BCA Reagent B. The cleaned cell solution was diluted 1:5 to achieve a concentration in the range of the assay, and EVs were measured as prepared. The samples and standards were mixed with WR at a ratio of 1:8 in a 96-well plate and placed on a shaker to ensure proper mixing. The well plate was then incubated at 37 °C for 30 min before measuring the absorbance at 562 nm. The absorbance of the BSA solution was plotted against the concentrations to achieve a standard curve and a standard curve equation. The amount of protein in the cell sample could then be calculated from the equation.

### 2.13 Western Blot

Cells (cleaned from FBS) and purified EVs were mixed with 2× Laemmli Sample Buffer and boiled for 5 min at 95 °C. To a 4–20% Mini-PROTEAN TGX Stain-Free Protein Gel, 2 μL of the cell (∼7 μg) and EV sample (∼5.8 ± 1.7 μg) was added, and 2.5 μL of all blue precision plus protein standard (ladder) was added. The gel was initially run at a lower speed (20 V) for samples to enter the gel, and after 15 min, the speed was increased to 120 V and ran for 45 min in a Mini-PROTEAN Tetra Vertical Electrophoresis Cell. The protein bands were then transferred to an iBlot 2 PVDF membrane in the iBlot 2 Gel Transfer Device and the membrane was cut into 5 pieces each one containing cells, EVs, and ladder. The membranes were then blocked with either 5% NFDM/TBST (for fibronectin), 5% FBS/TBST (for collagen and CD63), or 0.5% BSA/TBST (for calnexin and CD81) for 45 min. Then, the primary antibody was added; anticalnexin (rabbit), anticollagen (rabbit), anti-CD63 (mouse), anti-CD81 (mouse), and antifibronectin (rabbit) at 1/1000 and incubated overnight at 4 °C (except for antifibronectin which was incubated at RT for 1 h). The next day, the samples were washed 5 times with TBST and then secondary antibodies (Goat antirabbit igG (H + L)-HRP conjugation or Goat antimouse igG (H + L)-HRP conjugation) were added at 1/1000 and incubated for approximately 1 h. The samples were then washed 5 times with TBST and then washed in only TBS before being added to a Clarity Western ECL Substrate for 5 min before measuring the chemiluminescence in a ChemiDoc imaging system (Bio-Rad).

### 2.14 Proteomics

Isolated EV samples and cells (cleaned from FBS) were freeze-dried and sent to Proteome Center Tuebingen where the proteins were degraded with SDS, reduced by DTT, cleaned with S-trap cleaning, and digested with trypsin before loading approximately 500 ng of the sample onto the Thermo Scientific Q Exactive HF Orbitrap LC–MS/MS System and measured with a 60 min gradient, top7, sensitive method. Analysis was done using Max Quant version 2.2.0.0 against human database from UniProt (https://www.uniprot.org/). Proteins were then categorized according to their cellular locations, which can be seen in the Supporting Information Table S1. The different locations were: (1) cell membrane, (2) cytoplasm (cytoskeleton/motor), (3) Cytoplasm (Soluble), (4) ER/Golgi, (5) Extracellular (Secreted), (6) Lysosome, (7) Mitochondria, (8) Nucleus, and (9) Unknown. Many of the proteins are found at several locations. The classification was done using the subcellular location at UniProt (uniport annotion and GO annotation) (https://www.uniprot.org/).

### 2.15 Transmission Electron Microscopy (TEM)

TEM was performed on the BioVis platform of Uppsala University. A 5 μL drop of the sample was placed on a Formvar- and carbon-coated 200-mesh copper grid (Ted Pella). The excess solution was removed by blotting with a filter paper. The sample was briefly washed with MQ water and then directly contrasted with 2% uranyl acetate. Excess uranyl acetate was removed by blotting on filter paper. Images were acquired with a Tecnai G2 Spirit BioTwin transmission electron microscope (Thermo Fisher/FEI) at 80 kV with an ORIUS SC200 CCD camera and Gatan Digital Micrograph software (both from Gatan Inc./Blue Scientific).

### 2.16 Scratch Assay

IM-HCEpiCs were seeded in collagen-coated 48-well plates with a seeding density of 15 000 cells/cm^2^. The cells were left until approximately 90% confluency was achieved (2 days). Then, a 200 μL pipet tip was used to scratch the monolayer in a cross. The cells were washed twice with DPBS to remove debris and smoothen the edges. 250 μL of solutions containing 20 μg/mL (1 × 10^9^ particles), 100 μg/mL (1 × 10^10^ particles), or 200 μg/mL (2 × 10^10^ particles) EVs in Opti-MEM was added. Wells with only Opti-MEM were used as a negative control. Opti-MEM was used to avoid proliferation and look only at the healing of the scratched epithelium. Brightfield/phase contrast photographs were taken right after scratching and 24 h after the addition of EVs in Leica DMi8. Live/dead staining was performed on wells after 24 h to see the healing of the scratched epithelium and studied in a fluorescence microscope (Leica DMi8).

### 2.17 Release Studies

Corneal epithelial EVs were isolated and purified, as described above. The EVs were then stained using carboxyfluorescein succinimidyl ester (CFSE). One μL of CFSE (1 μL) was diluted in PBS and incubated 5 min at RT. The EVs were then added to the CFSE solution to reach a final concentration of 5 μM CFSE and the mixture was incubated for 25 min at 37 °C. After the incubation, the sample was then centrifuged at 1000 RCF in a 300 kDa Vivaspin 20 Centrifugal Concentrator using PBS to wash off excess CFSE (in total 6500 times diluted). Due to low fluorescence, the sample was stained an additional time following the same procedure but using a Amicon Ultra Centrifugal Filter 2 mL, 10 kDa MWCO, and centrifuging at 2000 RCF using PBS to wash off excess CFSE (in total 730 times diluted). The stained EV solution was measured in NTA, fluorescence spectroscopy, and confocal microscope.

Thiolated collagen was prepared as described elsewhere.^28^ A collagen/PEG hydrogel was prepared by mixing thiolated collagen with PEG-maleimide at 1:1 ratio between thiol and maleimide in a syringe mixing system.^27,29^ EVs at a concentration of 8.55 × 10^9^ particles/mL were added to the hydrogel and mixed. Approximately 125 mg of gel was added to each cuvette (which corresponds to ∼1.07 ×·10^9^ EVs) and left to cure the O/N with a lid at RT. Hydrogels without EVs were used as a negative control. The next day, 3 mL of PBS was added on top of the gels, and fluorescence of the solution on top of gels was measured on days 0, 1, 2, 10, 14, 20, 30, and 35. A calibration curve of fluorescently stained EVs was used to determine the percentage of release. Gels containing stained EVs were done in quadruplicates and gels without EVs were done in triplicates.

### 2.18 Collagenase Assay after Release Studies

After 35 days of incubation at RT, collagenase at a final concentration of 5 U/mL and 5 mM CaCl_2_ was added to the cuvettes with gels. The cuvettes were kept at 25 °C for the first 4 h and were then placed at 37 °C. On day 3, additional collagenase was added to a final concentration of 30 U/mL. The degradation was studied until the hydrogels were completely degraded, and photos were taken on days 1, 2, 3, and 6.

### 2.19 Confocal Microscopy and Fluorescence Recovery after Photobleaching

To investigate the mesh size of the gel, the distribution, and the diffusion of dextrans with different sizes, confocal microscopy and fluorescence recovery after photobleaching (FRAP) measurement were carried out. To carry out these measurements, gel discs were immersed into 1 mg/mL PBS solution of fluorescence isothiocyanate-labeled dextran with different molecular weights (4, 70, and 500 kDa). After 2 days, the samples were measured with a ZEISS LSM-700 fluorescence microscope (Oberkochen, Germany) using different setups for the different investigations.

The three-dimensional distribution of the nanoparticles in the gel matrix was carried out using silica nanoparticles (SiNPs) and the previously mentioned EVs. Nanoparticle suspensions were added to the gel precursor solution and mixed thoroughly to homogenize the mixture. The gels were kept under humid conditions to avoid drying and the release of the nanoparticles. The samples were investigated after 2 days using the previously mentioned ZEISS microscope.

To analyze the three-dimensional distribution of the FITC-dextrans and the nanoparticles in the gel matrix, a 25× water immersion objective was used, and Z-stack image acquisition was carried out. In each measurement, 40 images were taken with 1.1 μm steps in the *Z* direction about a 600 × 600 μm area from the edge and the bulk phase of the gel samples.

To measure the diffusion of the different FITC-dextrans, a 20× air objective was used. A 100 × 100 μm rectangular region of interest (ROI) was selected on a 600 × 600 μm image for bleaching. Six parallel measurements were carried out both in the gel matrix and in solutions. Bleaching was applied in the ROI for different time intervals to reduce the average intensity by 25% and recovery measured up to 95% of the original average intensity in the ROI. The data were analyzed by Frapsolver software (https://gitlab.com/jgernandt/frap-solver) earlier developed by us. The diffusion coefficients were obtained by fitting a rectangular FRAP pure diffusion implemented into the software.^30^

To track the EV and SiNP motion, time series measurement was carried out using the previously mentioned ZEISS microscope with a 20× magnification objective. In each measurement, 50 images were taken. To acquire the particle tracking data, ImageJ was used. The first brightness and contrast adjustment and background subtraction were applied by setting the rolling ball radius to 2 pixels. Afterward, particle tracking was carried out by using MTrackJ plugins.^31^ The snap feature was set to maximum intensity, and a 5 × 5 pixel snap range was used. The maximum distance to the start point was exported and plotted as a histogram using GraphPad Prism 8.

### 2.20 Image Processing and Analysis

Three-dimensional image construction and determining the distribution of the different FITC-dextrans between the gel matrix and the surrounding medium were done using Huygens Professional software (Hilversum, The Netherlands).

For the three-dimensional images, the Z-stack images were first deconvolved by using the deconvolution wizard of the Huygens Professional software (signal-to-noise ratio: 50, maximum iteration steps of 150). Afterward, the 3D images were constructed by using the maximum intensity projection (MIP) rendering option.

### 2.21 Calculation of the Polymer Volume Fraction in the Hydrogel

The polymer volume fraction of the hydrogel was calculated from the polymer mass fraction of 0.0206, as reported earlier,^28^ for shape-retaining hydrogels considering the density of collagen and water as 1.3 g/mL and 0.997 g/mL, respectively.

### 2.22 Fitting of the Theoretical Model

EV in vitro release profiles showing fraction released as a function of time were analyzed with the following expression describing the release from an initially homogeneous gel slab of thickness *l*, valid when the concentration is maintained equal to zero at the gel surface (sink condition)^32^

1 $$\frac{M_{t}}{M_{\infty }}=1-\sum_{n=0}^{\infty }\frac{8}{{\left(2n+1\right)}^{2}\pi^{2}}\exp\left\{-D{\left(2n+1\right)}^{2}\pi^{2}t/4l^{2}\right\}$$

where *M*_*t*_ is the amount of the diffusing substance released after time *t* and *M*_∞_ is the corresponding quantity after infinite time, and *D* is the diffusion coefficient. To account for a fraction of immobilized EVs in the gel, the function *f*_mob_ *M*_*t*_/*M*_∞_ was fitted to the release profiles, where *f*_mob_ is the mobile fraction. In the model fit, *l* was set to be equal to the experimentally determined gel thickness (1.26 mm).

Self-diffusion coefficients (*D*_g_) for FITC-labeled dextran diffusion probes measured with FRAP in the hydrogels were used to estimate the “mesh size” ( r̅ ) of the network by means of the Ogston–Amsden obstruction theory^33^

2a $$\frac{D_{g}}{D_{0}}=\exp\left\{-\frac{\pi }{4}{\left(\frac{R_{h}+R_{f}}{\mathrm{r̅}+R_{f}}\right)}^{2}\right\}$$

2b $$\mathrm{r̅}=\frac{R_{f}}{2\sqrt{\varphi /\pi }}$$

where *D*_0_ is the self-diffusion coefficient in an aqueous solution, *R*_h_ is the hydrodynamic radius of the diffusion probe, *R*_f_ is the radius of the polymer network fibers, φ is the volume fraction of the network. In the model, the diffusion probe is assumed to be spherical and the network is described as a random meshwork of randomly distributed straight fibers; r̅ is calculated as the largest radius of sphere that on the average can fit into the network structure without touching fibers.^34^ Equations 2a and 2b were used to calculate *D*_g_ as a function of *R*_h_ for *R*_f_ equal to 1 and 10 nm, respectively. In the calculation, the polymer network volume fraction in the hydrogel was set to 0.016, as determined experimentally, and *D*_0_ was calculated by means of the Stokes–Einstein equation

3 $$D_{0}=\frac{k_{B}T}{6\pi \eta R_{h}}$$

where *k*_B_ is Boltzmann’s constant, *T* is the temperature (307 K), η is the viscosity of water (7.34 × 10^–4^ N s/m^2^).

For comparison with a model describing the combined effect of obstruction and hydrodynamic interactions, the same calculation was made with the Clague–Phillips theory^35^

4a $$\frac{D_{g}}{D_{0}}={\left(1+\frac{2\alpha }{3}\right)}^{-1}\exp\left\{-\pi \varphi^{0.174\ln\left(59.6R_{f}/R_{s}\right)}\right\}$$

4b $$\alpha =\varphi {\left(\frac{R_{s}+R_{f}}{R_{f}}\right)}^{2}$$

The pre-exponential term in eqs 4a and 4b is the contribution from obstruction effects derived by Tsai and Streider,^36^ and the exponential term is from a regression analysis based on simulation results of hydrodynamic effects on spherical solute diffusion through a random network of cylindrical fibers.^33,35^

### 2.23 Viscoelastic Stress Relaxation

A dynamic oscillatory frequency sweep was performed on a strain-controlled ARES rheometer (TA Instruments, Sollentuna, Sweden) at 25 °C from 0.01 to 10 Hz at 0.267% oscillation strain amplitude using an 8 mm parallel plate stainless steel geometry. Oscillatory measurement was performed under a constant axial load of 0.1 N to prevent wall-slip. Continuous relaxation time spectrum, *H*(τ), was calculated from the dynamic storage and loss moduli, *G*′(ω) and *G*″(ω), by using the built-in nonlinear regression functions of the rheometer software (Trios version 5.4.0.300).^37^ Relaxation modulus *G*(*t*) was obtained from *H*(τ). The highest *G*(*t*) value was normalized to 1, and a normalized *G*(*t*) over time was plotted.

### 2.24 Statistical Analysis

The statistical analysis was performed in R using the “lme4”, “stats”, and “emmeans” packages. A simple general linear model was constructed followed by a post hoc test where the estimated marginal means (emmeans) and the standard error were calculated with a Bonferroni correction. Significant differences are shown by “*” in the graphs, “*” represents a *p*-value of <0.05, “**” a *p*-value of <0.01, and “***” a *p*-value of <0.001. Error bars in the graphs represent the standard deviation.

## 3 Results and Discussion

### 3.1 Isolation and Characterization of Corneal Epithelial Extracellular Vesicles

Corneal epithelial extracellular vesicles (CE-EVs) were isolated by treating a semiconfluent layer with a starvation medium (Opti-MEM) for 48 h to trigger EV production. After starvation treatment with Opti-MEM, one-third of the treated flasks were analyzed for the live/dead cell ratio using trypan blue staining, which showed 97–99% cell viability. Additionally, cell counts after treatment revealed a nearly 4-fold increase (378%) compared to initial seeding, indicating proliferation (in normal growth media) and robust survival (in the Opti-MEM) during the treatment. The medium was then collected and purified by tangential flow filtration (TFF), followed by concentration of the sample with an Amicon Ultra Centrifugal Filter. TFF was used for EV-purification instead of the more commonly employed ultracentrifugation (UC) due to the reported higher yield in TFF purification.^38^ Purified CE-EVs were stored in the fridge and used within 1–2 weeks or otherwise frozen at −20 °C in freezing media containing PBS with HEPES, Albumin, and Trehalose as described elsewhere.^39^

Nanoparticle tracking analysis (NTA) was then performed to determine the concentration and size of the CE-EVs. NTA was measured of samples before, during, and after purification to see how the size, distribution, and number of particles were affected (Figure S1). In Figure 1a, the size distribution of the purified CE-EVs (from 9 batches) is presented and the main peak for the samples was found to be in the range of 110 ± 12 nm which correlates to the size range of EVs.^15,16^ The size and morphology of CE-EVs were also studied by transmission electron microscopy (TEM) (Figure 1b). The size was found to be slightly smaller (around 60–70 nm) compared to NTA and aggregation could be observed.

**Figure 1:**
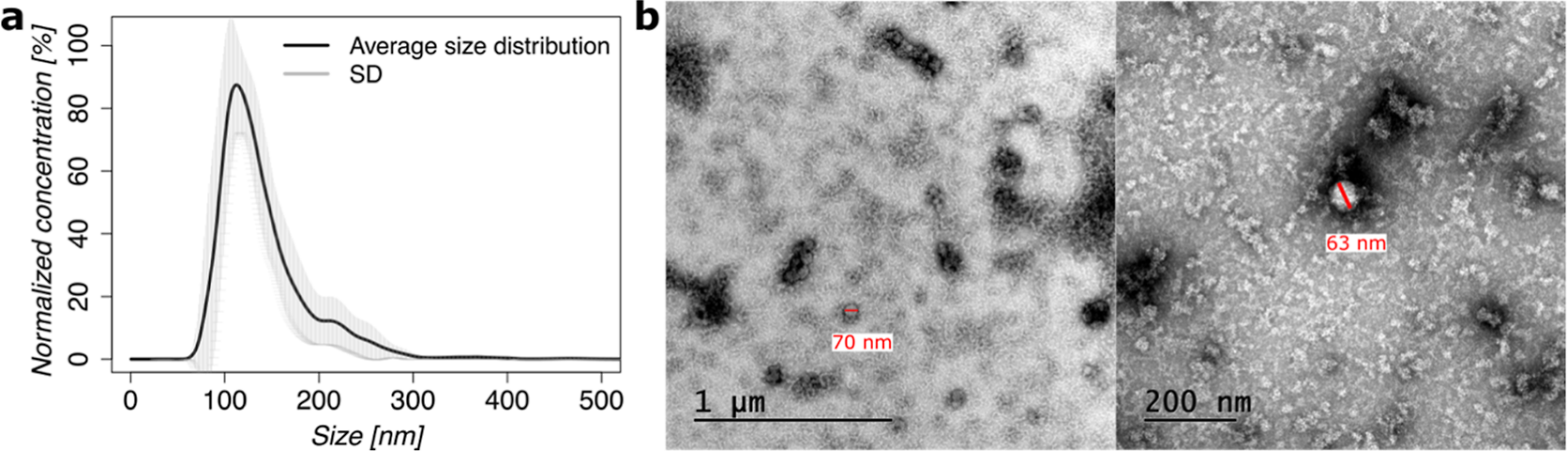
Size and morphology of CE-EVs after purification. (a) Nanoparticle tracking analysis (NTA) showing the average size distribution of the CE-EVs (for *n* = 9 batches). (b) TEM images of CE-EVs at different magnifications showing the size and shape of the CE-EVs.

### 3.2 Epitope Profiling of CE-EVs

To further characterize the CE-EVs, fluorescence-activated cell sorting (FACS) was performed to determine the surface markers on the CE-EVs. The complete FACS data are shown in Figures S2–S4, and the most expressed markers are shown in Figure 2a. For all batches (*n* = 8), the most common surface markers were the EV-specific markers CD63 (most expressed, set to 100%), CD9 (59.4 ± 24.4%), and CD81 (62.5 ± 10.2%).^40^ Aside from the EV-specific markers, the most expressed surface protein was CD29 (integrin β1) with 32.8 ± 13.8%. CD29 is part of many cell surface receptors which are responsible for the interaction of cells with ECM proteins.^41^ Moreover, in most of the batches, CD49E (integrin α5) was also highly expressed (average 32.6 ± 23.6%) which together with CD29 constitutes the fibronectin receptor (integrin α5β1).^41^ CD29 can constitute receptors with multiple other integrin α subunits that are not included in the MACSPlex EV kit, such as CD49C (integrin α3).^41^ Another highly expressed protein was the CD44 (17.9 ± 8.6%); a transmembrane cell surface receptor responsible for both cell–cell adhesion and cell–matrix interaction, which is usually upregulated after tissue injury.^42^ Further abundantly expressed surface markers were CD142 (17.6 ± 4.5%), CD40 (16.3 ± 3.9%), CD105 (14.1 ± 12.5%), CD146 (12.1 ± 12.6%), HLA-ABC (11.0 ± 6.5%), CD326 (7.4 ± 4.8%), MSCP (5.5 ± 5.1%), and HLA DRDPRDQ (4.8 ± 4.5%). These proteins are known to have crucial roles in cell adhesion and immunomodulation (Figure 2a).

**Figure 2:**
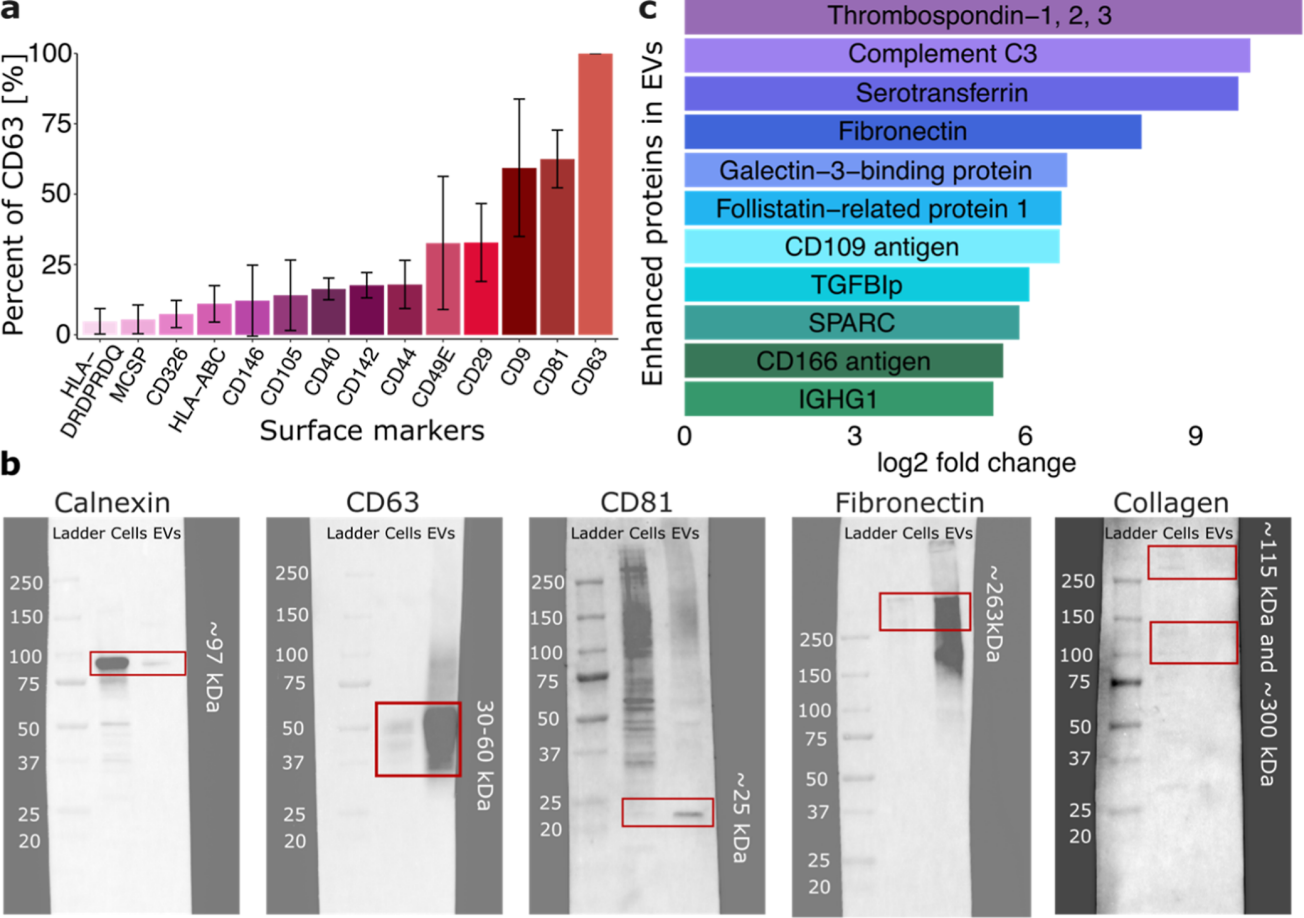
Protein markers demonstrated by (a) FACS with the MACSPlex kit demonstrating the surface markers of the CE-EVs, (b) Western blot showing the expression of calnexin, CD63, CD81, fibronectin, and collagen in CE-cells and CE-EVs, and (c) enhanced proteins in CE-EV samples compared to CE cells determined with LC–MS proteomics. Error bars in the graph represent the standard deviation of *n* = 8 batches.

For Western blot, proteomics, and scratch assay analyses, it was necessary to determine the total protein amounts in CE-EVs and CE-cells. This was done by using the BCA assay. For CE-EVs, the total amount of proteins was determined to be approximately 5 × 10^–9^ μg protein/particle and for CE-cells, it was approximately 7 μg/mL.

Western blotting was performed to study the expression of some of the proteins of interest (Figure 2b). CD63 and CD81 were investigated due to their EV-specific nature and to confirm the presence of these which was indicated from FACS. It was observed that both CD63 and CD81 were much more abundant in the CE-EVs compared to the CE cells (Figure 2b), supporting the presence of membrane-bound vesicles in the sample.^15,16^ Calnexin, on the other hand, is an endoplasmic reticulum (ER) protein used as a negative staining^43^ since it should not be present in the CE-EVs. As can be seen in Figure 2b, the CE cells expressed much higher levels of calnexin compared to the CE-EVs. The low levels of calnexin in the CE-EV sample could indicate low levels of residual cell debris. Furthermore, collagen type I and fibronectin were investigated since ECM proteins are considered to be important for corneal wound healing.^13^ Collagen type I was not very abundant in either CE cells or CE-EVs and the levels seemed to be similar in both, suggesting that collagen is not accumulated in the CE-EVs. However, fibronectin was significantly more abundant in the CE-EVs compared to the CE-cells, suggesting its importance. This is in agreement with the literature which suggests that fibronectin and other provisional ECM proteins are important in the first stages of wound healing in the cornea and that collagen type I is formed at a later stage of wound healing.^44^

### 3.3 Proteomics of CE-EVs

To study the overall protein composition of the CE-EVs, protein identification proteomics using LC–MS/MS was performed. All proteins found in the CE-EVs are presented in Table S1 and the proteins with the highest intensity-based absolute quantification (IBAQ) values are shown in Figure S5. Previous studies^22^ have investigated the protein content of CE-EVs and suggested that thrombospondin-1 (TSP-1), fibronectin, and other provisional extracellular matrix proteins are important components for corneal wound healing.^22^ Herein, we have studied the difference in protein abundance for CE-EVs and CE cells to assess which proteins have been enriched in the EVs and, therefore, indicate their importance as signaling molecules. The most enriched proteins are shown in Figure 2c and all enhanced proteins are presented in Figure S6. TSP-1, 2, and 3 were the most enriched proteins in the CE-EVs (1874 times). TSP-1 has been demonstrated to be highly expressed in the early stages of corneal wound healing and is known to activate the latent form of transforming growth factor β (TGF-β) which in turn can promote myofibroblast differentiation.^45–47^ TSP-2 has also been linked to corneal wound healing but is instead involved in the remodeling of the ECM at the later stages of healing by modulation of MMP-2 (72 kDa type-IV collagenase) and MMP-9 (92 kDa type-IV collagenase) which were both also found in the CE-EVs (Table S1).^46,47^ The second most enriched protein was the Complement C3, which is a key component of the complement system and has a role in inflammation induction and immune cell recruitment.^48^ The complement system is a part of the immune defense, which not only protects the body against foreign pathogens but has also been suggested to play a significant role in wound healing.^49^ In the CE-EVs, complement C3 was enriched 993 times (Figure 2c), and several other components of the complement system (Complement C1r, C 1s, factor I, factor H, factor B, C4-A, and C4-B) were exclusively found in the CE-EVs (Table S1), suggesting their importance for wound healing. This is supported by the literature that has demonstrated complement C3 to be involved in tissue regeneration^48^ (e.g., in the retina of chicks^50^). Moreover, serotransferrin was enriched 860 times in CE-EVs (Figure 2c) and is an important iron transporter in the body. Iron is involved in several biological processes such as cell proliferation and proper functioning of immune cells, both of which are processes involved in corneal wound healing^51^ and could potentially explain the abundance of serotransferrin in the CE-EVs. In accordance with previous studies of corneal epithelial EVs^22^ and with the Western blot results, fibronectin was highly enriched (264 times) in the CE-EVs compared to CE cells according to proteomics (Figure 2c). Fibronectin has been proven important for corneal wound healing acting as a temporary scaffold for migrating epithelial cells.^41^ Another enhanced protein in the CE-EVs was the galectin-3 binding protein (LGALS3BP) (enriched 106 times; Figure 2c). LGALS3BP has been demonstrated to enhance cell adhesion through integrin-mediated interactions (specifically α5β1).^52^ Follistatin-related protein 1 (FSTL1), which was enriched 99 times in the CE-EVs, is known to play a role in the regulation of cell proliferation, differentiation, and migration in both physiological and pathological processes.^53^ However, the exact and direct role of this protein in corneal wound healing is not well understood. FSTL1 is a glycoprotein that belongs to the family of secreted proteins acidic and rich in cysteine (SPARC).^54^ SPARC also called osteonectin (ON) was enhanced 59 times in CE-EVs compared with CE cells (Figure 2c). SPARC has been demonstrated to inhibit epithelial cell proliferation by binding to TGF-β receptors.^55^ It has also been demonstrated that SPARC is strongly expressed during the middle and later stages of corneal wound healing to facilitate remodeling and that SPARC increases the wound closure rate.^56,57^ Another protein that regulates TGF-β signaling is CD109. In contrast to TSP-1, CD109 has an antagonistic effect on TGF-β signaling and inhibits extracellular matrix deposition by binding to TGF-β.^58^ CD109 was enhanced 97 times in the CE-EVs compared to CE cells (Figure 2c), suggesting the need for a counterbalance of TGF-β signaling to avoid excessive extracellular matrix (ECM) deposition. Transforming growth factor beta-induced protein (TGFBIp) was enhanced 67 times in CE-EVs compared to CE cells (Figure 2c) and it is known to be highly abundant in the cornea and highly expressed by epithelial cells.^59^ TGFBIp has been demonstrated to be upregulated in case of corneal damage in rabbits^60^ and some studies suggest that TGFBIp promotes corneal wound healing by increased cell migration, adhesion, and proliferation.^61,62^ CD166 (enriched 49 times in CE-EVs compared to CE cells) (Figure 2c), also known as an activated leukocyte cell adhesion molecule (ALCAM), is known to be a marker for corneal stromal stem cells and is involved in ocular regeneration.^63^ Immunoglobulin heavy constant gamma 1 (IGHG1), which is enriched 43 times in the EVs (Figure 2c), is part of the immunoglobulin G1 (IgG1) antibody. Several other components for immunoglobulins (such as IgG2, IgG3, IgA1, and IgA2) were found exclusively in CE-EVs suggesting that the adaptive immune system is important for wound healing. IgG1 (and IgG3) has also been shown to activate the complement system, which plays an important role in wound healing.

Some of the surface markers detected in the FACS analysis could be confirmed with the proteomics data; CD63, CD81, and CD142 (Tissue factor) are found exclusively in CE-EVs (Table S1), whereas the CD146/MUC18 (enriched 2.8 times) and CD44 (enriched 4.8 times) were found both in CE-EVs and CE-cells but were enriched in CE-EVs (Table S1). On the other hand, HLA-ABC is slightly altered in the CE-EVs compared to CE cells (ratio 0.42 for HLA-A and C and 0.84 for HLA-B, Table S1). Moreover, the components for the fibronectin receptor (α5β1) were also present and slightly enriched in the CE-EVs (∼2 times) (Table S1). Integrin β1 is known to be important during corneal wound healing for promoting the cellular attachment of migrating cells to the provisional matrix.^41^ Additionally, several other integrins, such as α3, β6, αv, and α6, were identified in the proteomics data (Table S1). Integrin α3 (CD49C) (enhanced 1.34 times, Table S1) together with integrin β1 (CD29) (enhanced 1.8 times, Table S1) constitutes the integrin α3β1 which is a receptor for laminin, fibronectin, collagen, and TSP-1.^41,64^ Furthermore, the integrins αv and β6, which were both found in the CE-EV sample (Table S1), together make a receptor that can also bind fibronectin.^65,66^ The integrin αvβ6 is exclusively found on epithelial cells^65,66^ and previous studies have shown the importance of the integrin αvβ6 receptor for corneal wound healing by its activation of TGF-β.^67^ Additionally, several subunits of laminin were found, some of which are exclusively present in CE-EVs (Table S1), and together can constitute laminin 411 and laminin 511; the latter is known to be important at the initial stage of corneal epithelial regeneration.^14^ Similarly, several subunits of collagen types I, V, and VI were found exclusively in CE-EVs (Table S1). Collagen VI has been shown to promote adhesion and spreading of corneal fibroblasts in chicks^68^ and in rabbits, it is increased during corneal wound healing and is suggested to be important for the formation of the corneal lamellar structure.^69^

Calnexin was also detected in the proteomics data; however, its ratio in CE-EVs compared with CE cells was 0.029. This significant reduction in CE-EVs is consistent with the Western blot results (Figure 2b and Table S1).

Corneal healing is a highly orchestrated process involving intricate intercellular communication between epithelial cells, stromal keratocytes, and endothelial cells to restore tissue integrity and transparency.^45^ Extracellular vesicles (EVs) serve as critical mediators in this communication network, transferring a diverse cargo of proteins, lipids, and nucleic acids between cells.^15,16^ Our comprehensive analyses of CE-EV proteins using FACS, Western blot, and proteomics, along with the assessment of their relative enrichment compared with CE-cells and integration with the current literature, illuminate several plausible mechanisms through which CE-EVs can promote corneal regeneration. Rapid re-epithelialization is crucial for restoring the cornea’s barrier function, and CE-EVs appear well equipped to facilitate this process. Effective epithelial cell migration requires dynamic regulation of cell adhesion to the underlying matrix and neighboring cells. Our proteomic analysis revealed that CE-EVs are enriched with proteins known to mediate these interactions, such as fibronectin (Table S1),^60^ which acts as a provisional matrix promoting epithelial cell migration and attachment, as demonstrated in earlier studies.^41,45^ CE-EVs also contain integrin subunits (e.g., β1, α3, α5, Table S1)^41^ and their ligands, which are crucial for cell movement and maintaining epithelial sheet integrity during migration.^41,70^ For instance, integrin α5β1 facilitates movement on the fibronectin-rich matrix, while α3β1 mediates cell–cell and cell–ECM adhesion.^41,70^ Transforming growth factor beta-induced protein (TGFBIp/Big-h3), identified in CE-EVs (Table S1),^60^ directly promotes adhesion via the α3β1 integrin.^70^ Furthermore, other enriched proteins like LGALS3BP (Table S1) and TSP-1 (Table S1) mediate adhesion through interactions with integrins and other receptors.^41,52,64^ CE-EV cargo might also influence the necessary disassembly of hemidesmosomes (involving the α6β4 integrin) for migration initiation.^41^ Beyond adhesion and migration, CE-EVs contain factors modulating the balance between cell proliferation and differentiation required to restore the epithelial thickness. For example, SPARC, enriched in CE-EVs (Table S1), can inhibit epithelial proliferation, partly by stimulating TGF-β signaling,^55^ while factors promoting adhesion indirectly support proliferation once the defect is covered. Communication between the epithelium and stroma via EVs becomes particularly important following injuries that compromise the epithelial basement membrane, allowing EVs direct access to stromal keratocytes.^45^ Stromal wound healing involves the transformation of quiescent keratocytes into activated fibroblasts and potentially myofibroblasts, which are crucial for wound contraction and initial matrix deposition. This process is heavily regulated by TGF-β signaling.^45^ CE-EVs can influence this pathway significantly. TSP-1, highly enriched in CE-EVs (Table S1), is a major physiological activator of latent TGF-β1,^45,55,64^ and its delivery via CE-EVs could directly promote myofibroblast differentiation in the stroma. Concurrently, CE-EVs carry proteins that modulate TGF-β activity; SPARC can stimulate TGF-β signaling,^55^ while CD109 (Table S1) acts as a negative regulator.^58^ This balance within CE-EVs could be crucial for controlling myofibroblast activity and limiting fibrosis. CE-EVs also contribute to ECM deposition and remodeling, both directly by carrying components like fibronectin and collagen fragments (Table S1)^60^ and indirectly through modulators like TGFBIp,^60,70^ TSP-1,^64^ LGALS3BP,^52^ and SPARC,^55^ often via TGF-β pathways. MMPs or regulators like TSP-1 within CE-EVs may also contribute to later-stage matrix remodeling (Table S1).^64^ Finally, wound healing involves controlled inflammation, and CE-EVs contain factors like Complement C3 and LGALS3BP (Table S1), that modulate immune cell activity, potentially influencing the resolution phase.^52^ In summary, CE-EVs encapsulate a complex repertoire of bioactive molecules, including adhesion proteins, ECM components, signaling molecules, and their modulators, that collectively promote corneal wound healing by facilitating epithelial migration and adhesion while also modulating stromal keratocyte activation, ECM deposition, and remodeling, primarily through intricate interactions with integrin and TGF-β signaling pathways. The enrichment of specific factors like TSP-1, fibronectin, LGALS3BP, SPARC, and CD109 within CE-EVs, as revealed by our analyses (Table S1), highlights their targeted role in orchestrating the multifaceted cellular responses required for successful corneal regeneration (Figure S7).^41,45,52,55,58,60,64,70^

### 3.4 Efficacy of CE-EVs in Healing the Corneal Epithelium

To further evaluate the potential of CE-EVs in corneal epithelial wound healing, an in vitro wound healing assay (scratch assay) was carried out. Human corneal epithelial cells were grown on a collagen-coated surface to form a confluent monolayer and then scratched using a pipet tip to create a wound mimic. Then, CE-EVs in different concentrations (20, 100, and 200 μg/mL) were added and left to incubate for 24 h before studying the closure of the scratched area (Figures 3a and S8). All CE-EV concentrations showed significantly higher healing compared with the negative control (Opti-MEM) (Figure 3b). However, the results for 20 μg/mL were more varying (66.8 ± 8.3%) and significantly lower (*p* <0.05) than 100 μg/mL (78.8 ± 3.8%). Both 100 μg/mL and 200 μg/mL (72.3 ± 4.7%) showed significantly higher (*p* <0.001) healing compared to the control (55.8 ± 1.9%) but not significantly different from each other. The data indicate that higher concentrations of CE-EVs enhance healing up to a certain threshold, beyond which no additional benefits are observed. This finding highlights the potential for scale-up and GMP production for future clinical applications, as the optimal effective concentration can be achieved relatively easily and is suitable for integration with a delivery matrix.

**Figure 3:**
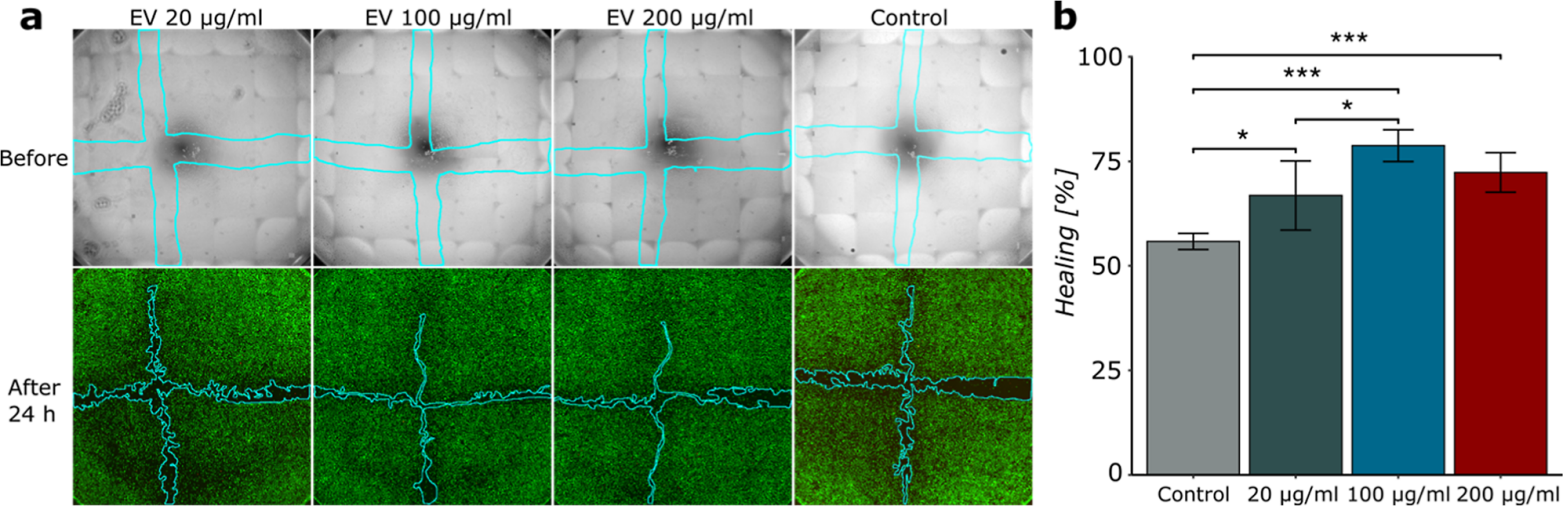
(a) Scratch assay of human corneal epithelial cells and (b) results of healing percentages for each group of the scratch assay. Error bars in the graph representing the standard deviation of *n* = 5 replicates. Significant differences are shown by “*” in the graphs, “*” representing a *p*-value of <0.05, “**” a *p*-value of <0.01, and “***” a *p*-value of <0.001.

### 3.5 Sustained and On-Demand Delivery of CE-EVs Using the Hydrogel Matrix

EV delivery to the eye presents several significant challenges. Topical delivery methods often face rapid clearance due to blinking and tear production, whereas systemic delivery approaches are unsuitable due to cornea being immune-privileged, making localized delivery methods more crucial.^51^ Our earlier developed cross-linked collagen hydrogel showed promise in corneal perforation due to its injectable and adhesive nature.^28^ Furthermore, due to the shape-retaining nature of this hydrogel and the cytocompatibility of the cross-linking reaction, it would be possible to use such a hydrogel for in situ molding of corneal defects. Additionally, since these hydrogels can be stored in buffers without swelling and allow the proliferation of corneal epithelial cells, they are potentially useful as corneal implants during Deep Anterior Lamellar Keratoplasty (DALK). Sustained delivery of EVs during corneal perforation, in situ molding of corneal defects, and keratoplasty using corneal implants would be highly desirable. However, a prerequisite would be the wide range of delivery time scales; a perforation would require a relatively fast-to-moderate release rate as the hydrogel was supposed to act only as a temporary scaffold lasting over a few weeks,^28^ an in situ molding of stromal defects would require a release time over a few months as stromal remodeling is slow, and complete stromal regeneration after keratoplasty with a hydrogel stromal substitute could take even longer.^21^ Although it is impossible to mimic the exact in vivo scenario in vitro and nearly impossible to achieve all of these release time-scales through one hydrogel, it would be highly valuable if the release time-scales could be as broad as from days to years. For this purpose, we further evaluated the release of encapsulated CE-EVs from our previously reported collagen hydrogel as well as the distribution and mobility of these EVs inside the hydrogel matrix.

To better assess the release kinetics of EVs from the hydrogel matrix and to make fluorescence microscopy possible, EVs were fluorescently labeled. This was achieved by covalently attaching a fluorescent dye, carboxyfluorescein succinimidyl ester (CFSE), to the protein cargos of the EVs. Such fluorescence detection of released CE-EVs from the hydrogel can provide a more accurate quantitative assessment compared to NTA as shredded polypeptide fragments released from the cross-linked hydrogel can potentially appear as particles during NTA, leading to misinterpretation of the data.

The release experiment is depicted schematically in Figure 4a. To mimic a more in vivo scenario, a collagenase degradation step was also included (Figure 4a). EV release primarily through diffusion was monitored and was found to plateau at 36 ± 5% after 10 days (Figure 4b). Following this initial phase, the remaining EVs can only be released via an on-demand enzymatic degradation of the hydrogel, closely mimicking the in vivo scenario where host matrix metalloproteinases (MMPs) gradually remodel the hydrogel matrix. The cumulative EV release is presented in Figure S9 and was found to be within a range similar to that observed in the scratch assay. Notably, the incorporation of CE-EVs neither interferes with the hydrogel cross-linking mechanism nor alters its viscoelastic properties; thus, it would be feasible to encapsulate varying amounts of CE-EVs as required for potential in vivo studies in the future. The enzymatic degradation of the hydrogels can be seen in Figure S10.

**Figure 4:**
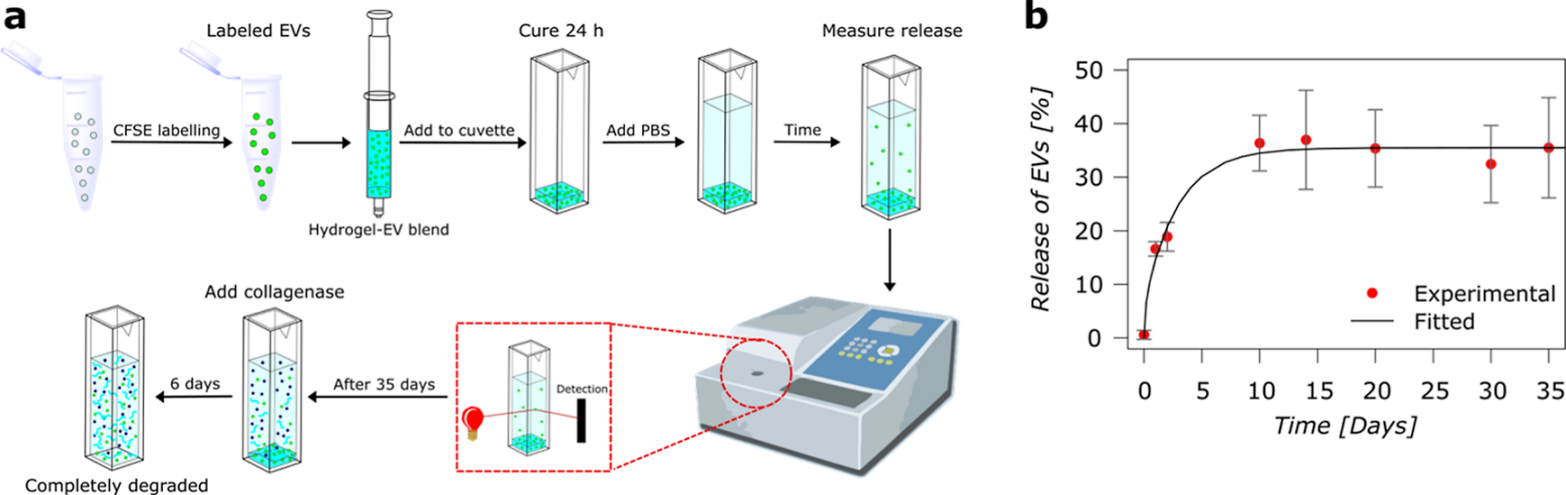
EV release. (a) Schematic of the EV release through diffusion and then after 35 days through collagenase degradation. (b) Release profile and fitted model of EVs from the hydrogel through diffusion.

The release profile was fitted to a mathematical 1D-diffusion model,^32^ yielding an empirical self-diffusion coefficient (empirical *D*_g_) of 2.5 μm^2^/s for encapsulated EVs in the hydrogel matrix (Figure 4b).

To further investigate the hydrogel network structure and its influence on EV distribution and diffusion within the hydrogel network, fluorescently labeled EVs and silica nanoparticles (SiNPs) of similar sizes were encapsulated in the hydrogel. Several aggregates with significantly larger sizes can be seen in the 3D images (Figure 5a). This aggregation can be induced by the interaction with the hydrogel matrix since neither the NTA nor the fluorescent microscopy in PBS showed similar structures. However, the majority of both EVs and SiNPs were found to be evenly distributed within the hydrogel and not released into the surrounding solution within the microscopy time scale (Figure 5a).

**Figure 5:**
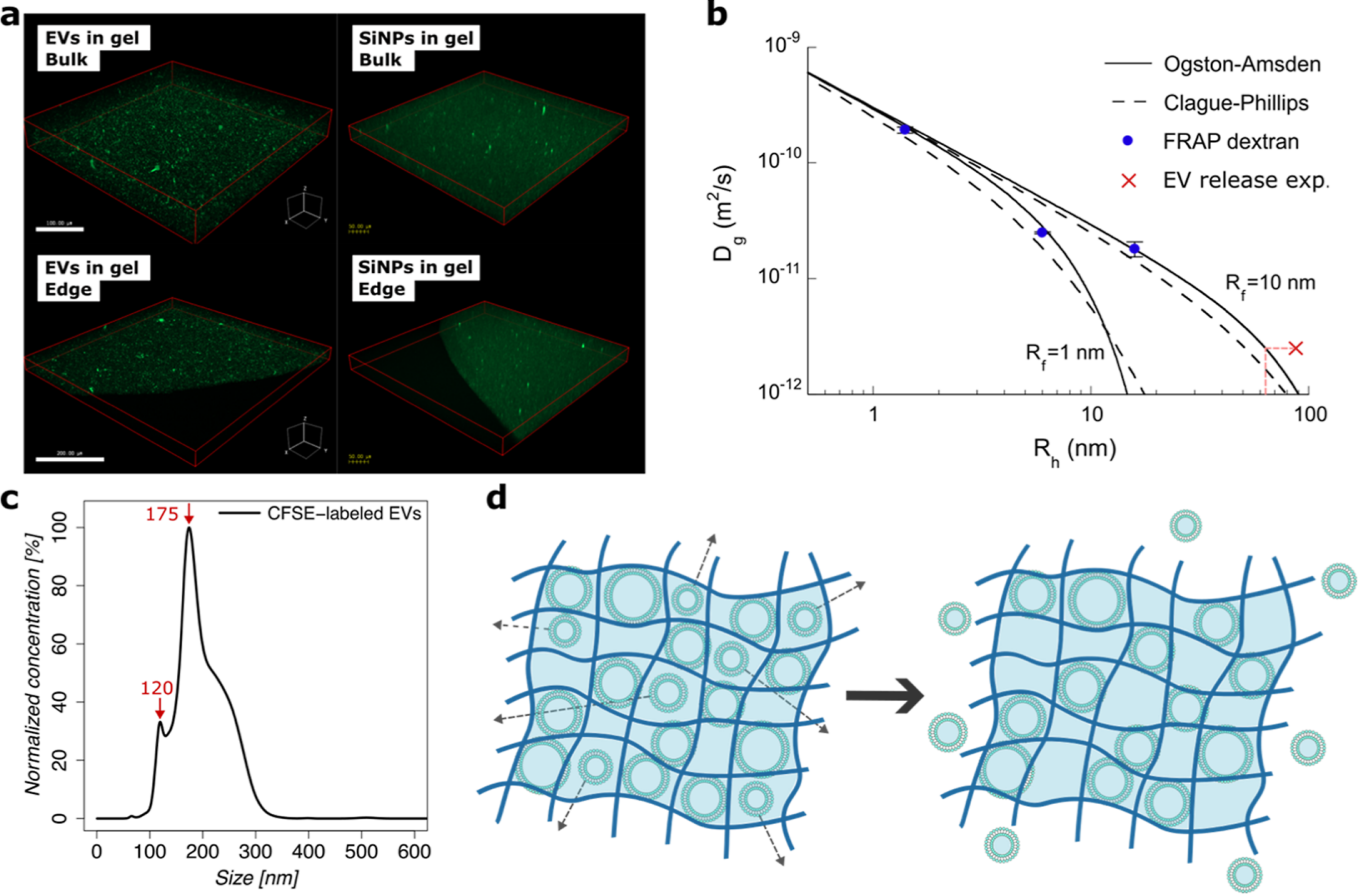
EVs in the hydrogel. (a) Confocal images of extracellular vesicles (EVs) and Silica nanoparticles (SiNPs) in the hydrogel showing both bulk phase and the edge, (b) fitting of Ogston–Amsden and Clague–Philips diffusion models for the hydrogel network from experimentally determined self-diffusion coefficients (*D*_g_) of various probes within the hydrogel assuming two different collagen fiber radii (*R*_f_) and position of the empirical self-diffusion coefficient obtained from the EV-release experiment, (c) size distribution from the nanoparticle tracking analysis (NTA) of CFSE-labeled EVs, and (d) schematic of the EV-release theory where smaller particles can diffuse out, while larger particles are obstructed by the hydrogel network.

To understand better the release mechanism of the EVs and the effect of the solute size on the diffusion parameters, Fluorescent Recovery after Photobleaching (FRAP) measurements were conducted using FITC-dextrans of varying molecular weights (4, 70, and 500 kDa). The distribution of these dextrans within the hydrogel and at the gel–solution interphase is shown in Figure S11, with two-dimensional images taken in fluorescence and merged (brightfield and fluorescence) channels. Additionally, 3D images constructed through z-stack analyses illustrated dextran distribution inside the hydrogel that shows each dextran probe is homogeneously distributed in the hydrogel matrix which is crucial to interpret the diffusion data acquired by FRAP (Figure S12). The diffusion coefficients determined via FRAP were 232 ± 12, 64.6 ± 0.5, and 36.7 ± 2.7 μm^2^/s for 4, 70, and 500 kDa dextrans, respectively, with reported hydrodynamic diameters of 1.4, 6.0, and 15.9 nm in an aqueous solution.^71^

Utilizing the dextran probe diffusion data, the mesh size of the hydrogel was estimated to be 14 and 140 nm corresponding to a collagen fiber radius of 1 and 10 nm, respectively, using the Ogston–Amsden model of diffusion in hydrogel networks (Figure 5b).^33,34^ The Clague–Philips model, which includes combined effects of obstruction and hydrodynamic interactions, gave similar results (Figure 5b).^35,36^ Moreover, the empirical *D*_g_ of the encapsulated EVs was found to be lying slightly outside of the fitted Ogston–Amsden model curve (Figure 5b). However, if this empirical *D*_g_ is moved onto the fitted Ogston–Amsden model curve, the radius of the released solute should have been ∼60 nm (Figure 5b), which is in very close agreement with the minor peak observed in the NTA of the labeled EVs (Figure 5c). To further validate the assumption of rigid fibers in the Ogston–Amsden model, a viscoelastic stress–relaxation experiment was performed which revealed no major decay in the normalized relaxation modulus over time (Figure S13). This modeling revealed a physical obstruction effect exerted by the hydrogel network on EVs, resulting in the preferential release of smaller particles through diffusion (Figure 5d). To support this hypothesis, we tracked the EV motion both in PBS and in the hydrogel matrix and compared the results with the silica nanoparticle (SiNP) motion in the hydrogels (Supporting Information Videos 1, 2, and 3). In Figure S14, the distribution of the maximum distance to the starting point of the particle is presented. These results show that the EVs are able to move freely in PBS, while this mobility is significantly reduced when the EVs are incorporated into the hydrogel matrix. By comparing the EVs with the SiNPs, we can observe that the maximum distances from the start point are slightly smaller in the case of SiNP compared to the EVs and only one population can be observed (Figure S14). On the other hand, the distribution graph of the EVs represents two populations (Figure S14). These data can support the EV-release data and explain why only 36% of the encapsulated EVs were able to be released from the hydrogel matrix through diffusion. The integration of quantitative assessment techniques and theoretical modeling enhances our understanding of EV-release dynamics and thereby facilitates the design of future ocular drug delivery platforms.

In vivo degradation dynamics of biomaterials within the corneal environment are largely governed by host enzymatic activities. While our collagenase assay indicates the hydrogel’s susceptibility to enzymatic breakdown, the process following implantation involves intricate host responses, including cell infiltration and the localized secretion of various enzymes, primarily matrix metalloproteinases (MMPs), which orchestrate tissue remodeling.^72^ Key MMPs implicated in corneal wound healing and matrix turnover include gelatinases (MMP-2, MMP-9), collagenases (MMP-1, MMP-8, MMP-13), stromelysins (MMP-3, MMP-7), and membrane-type MMPs (MT1-MMP/MMP-14).^73–76^ The activity profile of these MMPs varies significantly throughout the healing phases; for instance, MMP-2 and MMP-9 levels often peak during early epithelial migration (within hours to days postinjury), whereas others like MMP-8 and MT1-MMP can remain elevated for longer periods, contributing to sustained remodeling.^74,75^ This remodeling process is necessary for the integration of the implant and regeneration of the native tissue and often spans weeks to months and even years. Our earlier studies involving collagen and peptide-based implants demonstrated successful integration and tissue remodeling occurring over periods ranging from 3 to 12 months or even longer in vivo.^21^ Therefore, the enzymatic degradation susceptibility observed in our in vitro assay, though a simplified system, is consistent with the hydrogel’s potential for gradual resorption and replacement by the host tissue in vivo, mediated by endogenous MMP activity during the essential long-term remodeling phase of corneal regeneration. Furthermore, the observed biphasic release profile, initial diffusion-limited release over 10 days followed by the potential for subsequent release upon enzymatic matrix breakdown, suits well with the expected in vivo scenario involving early host cell integration followed by slower, MMP-mediated implant remodeling.

## 4 Conclusions

In conclusion, this study presents the first comprehensive characterization of corneal epithelial extracellular vesicles (CE-EVs) and provides significant insights into their role in corneal wound healing. By elucidating the interactions between various signaling proteins within the CE-EVs, we have advanced our understanding of the corneal wound healing pathway. Our findings demonstrate the potent efficacy of CE-EVs in promoting corneal epithelium wound healing in vitro and, therefore, highlight their potential as a therapeutic tool.

Moreover, we have addressed critical challenges in corneal drug delivery by developing a hydrogel-based delivery system that offers a broad time span for release. This system initially follows diffusion-controlled release, transitioning to an enzymatic (collagenase) digestion-controlled release on demand. Such release system is also a close mimic of the in vivo scenario where drug release occurs through the diffusion at the beginning followed by enzymatic degradation of the implant during the implant-remodeling phase. Our study provides a physical understanding of the release mechanisms at a microscopic level, supported by quantitative evaluation and microscale insights into EV diffusion within the hydrogel matrix. Utilizing established physical diffusion models, such as the Ogston–Amsden and Clague–Phillips models, we have achieved a detailed microscale understanding of these processes.

This foundational knowledge paves the way for the development of advanced hydrogel platforms tailored for EV release that accommodate diverse medical applications with varying release profiles. We believe these findings would equip researchers with a robust chemistry toolset to design hydrogels with predictable and controllable EV-diffusion and -release characteristics, thereby broadening the scope of therapeutic applications for extracellular vesicles in regenerative medicine.

## Supplementary Material









## Notes

Nanoparticle tracking analyses, FACS, proteomics data, interactions of proteins, scratch assay, cumulative EV release, collagenase degradation of hydrogels, confocal microscope images, viscoelastic stress relaxation, tracking histograms, and list of proteins identified by proteomics (PDF)
Supplementary video of EV in hydrogel (AVI)
Supplementary video of EV tracking in PBS (AVI)
Supplementary video of nanoparticle tracking in hydrogel (AVI)
