# Supplementary material for: Hydrogel-Mediated Sustained Delivery of Corneal Epithelial Extracellular Vesicles: A Strategy for Enhanced Corneal Regeneration
Source: ACS Omega. 2025 Aug 14;10(33):37081–95. doi: 10.1021/acsomega.5c01135 (PMC12391988; doi:10.1021/acsomega.5c01135)
Supplement: Supplementary file 1 [file ao5c01135_si_001.pdf]

Supporting Information for

Hydrogel-Mediated Sustained Delivery of Corneal Epithelial Extracellular Vesicles: A Strategy for Enhanced Corneal Regeneration

Jenny Rosenquist Lybecker,[†] *Ann Van de Ven,*[†] *Ken Braesch-Andersen,*[†, ‡] *David Juriga,*[#] *Norein Norein,*[†] *Per Hansson,*[#] *and Ayan Samanta*[*, †]

[†]Macromolecular Chemistry, Department of Chemistry – Ångström Laboratory, Uppsala University, Box 538, 751 21 Uppsala, Sweden

[‡]Thoracic Surgery, Department of Surgical Sciences, Uppsala University, 751 85, Uppsala, Sweden

[#]Pharmaceutical Physical Chemistry, Department of Medicinal Chemistry, Uppsala University, Box 574, 751 23 Uppsala, Sweden

*Corresponding author: ayan.samanta@kemi.uu.se

Page S1. This page

Page S2-S14. Figures S1-S14

Page S15. Table S1

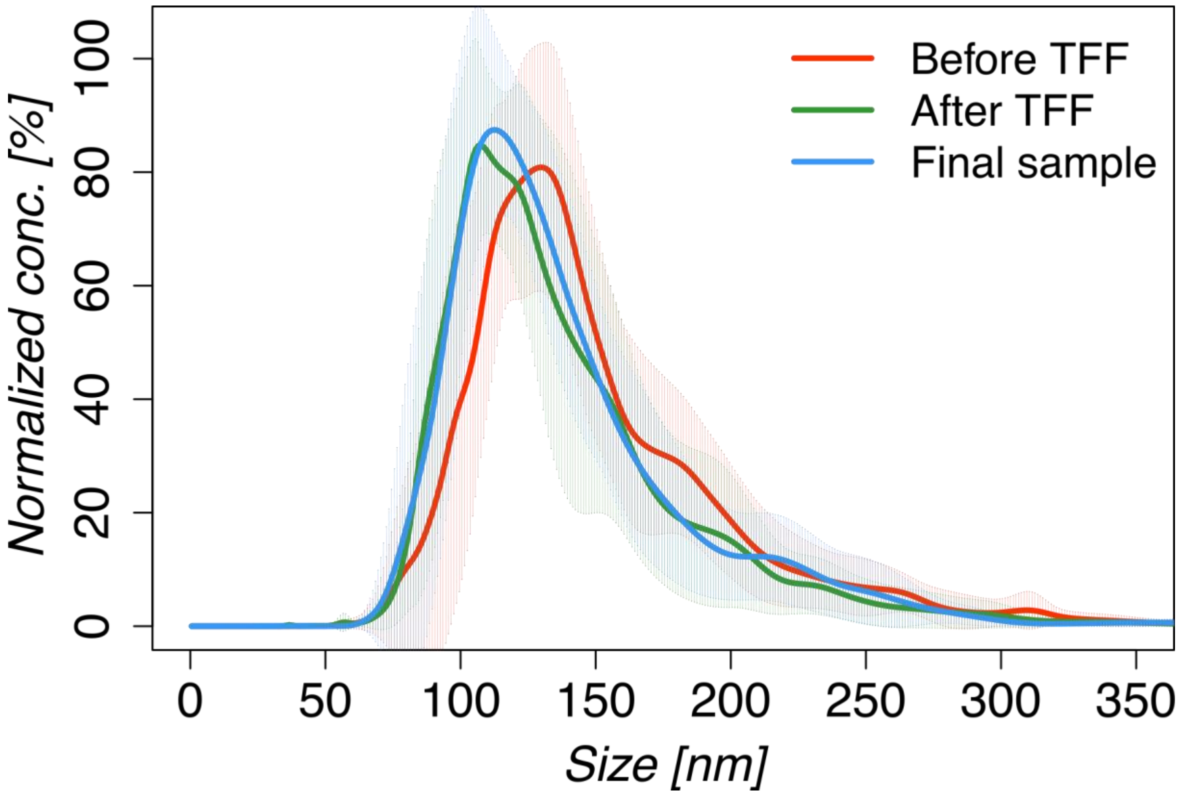

Figure S1. Overview of nanoparticle tracking analyses (NTA) of isolated (before TFF), purified (after TFF), and concentrated (final sample) extracellular vesicles.

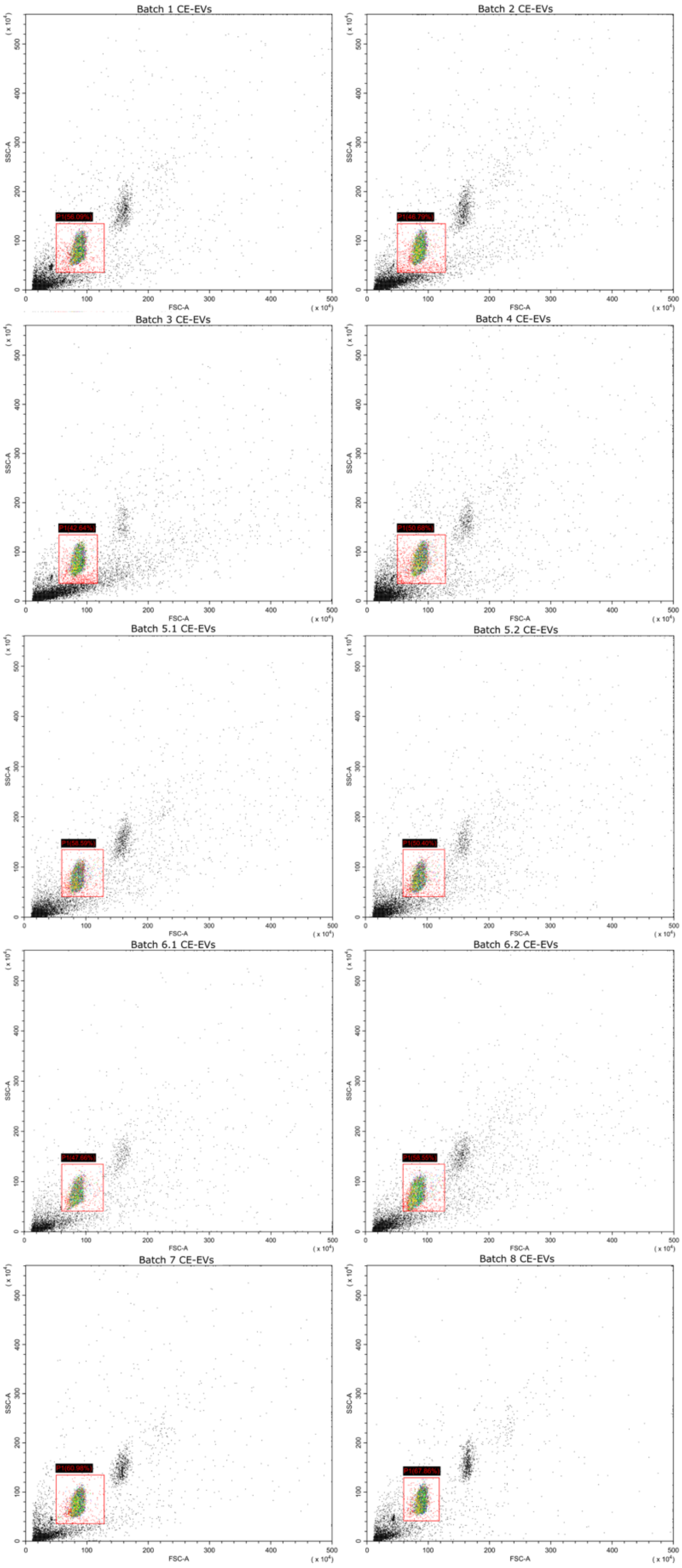

Figure S2. FACS scatter plots (with all events) for the 8 batches. The selected area P1 contains the EVs while smaller particles are debris and bigger sizes represent aggregates.

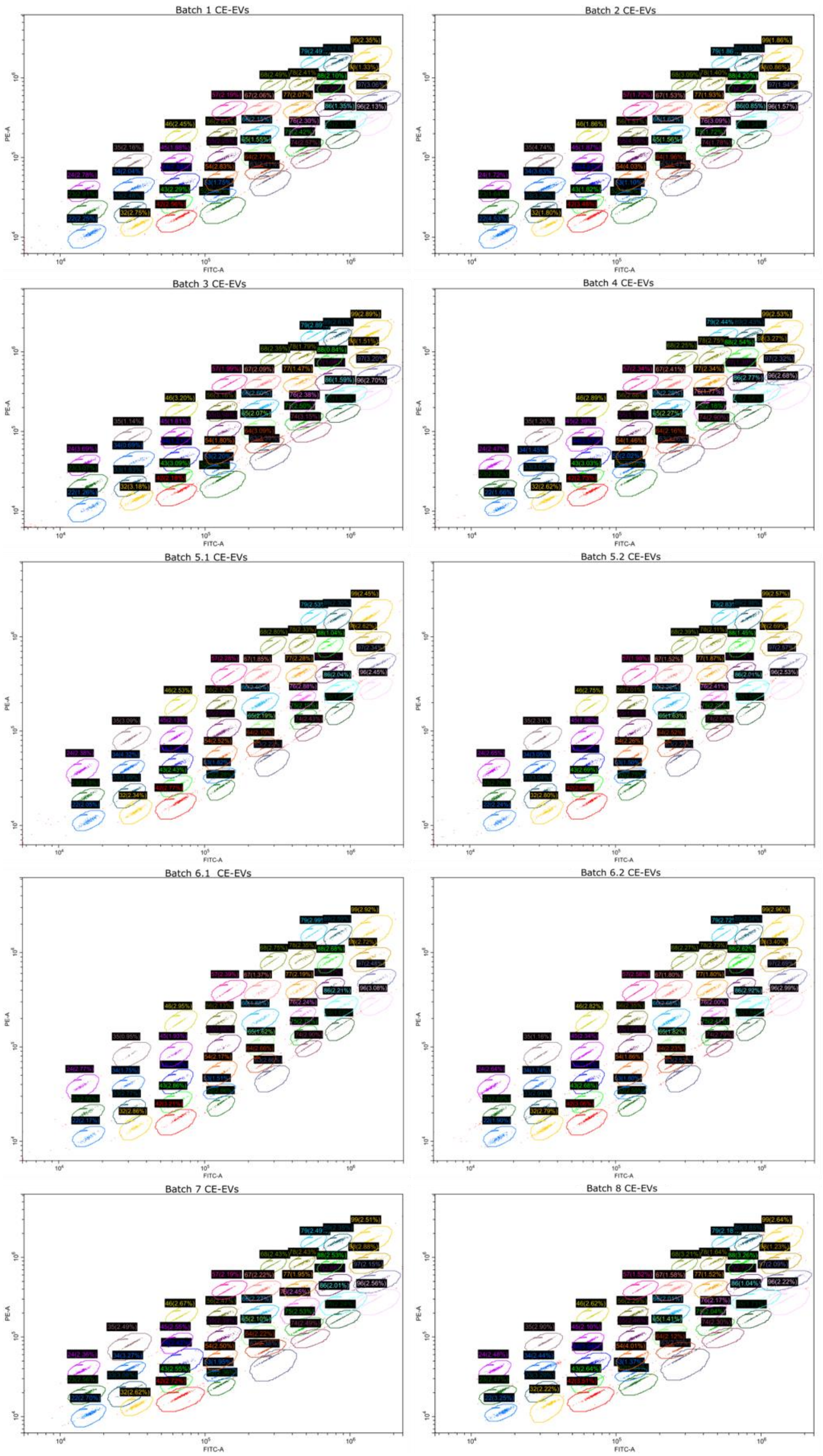

Figure S3. PE (phycoerythrin) vs FITC (fluorescein isothiocyanate) signal for the P1 area showing the presence of the 39 capture beads of the MACSPlex kit for all batches (n=8).

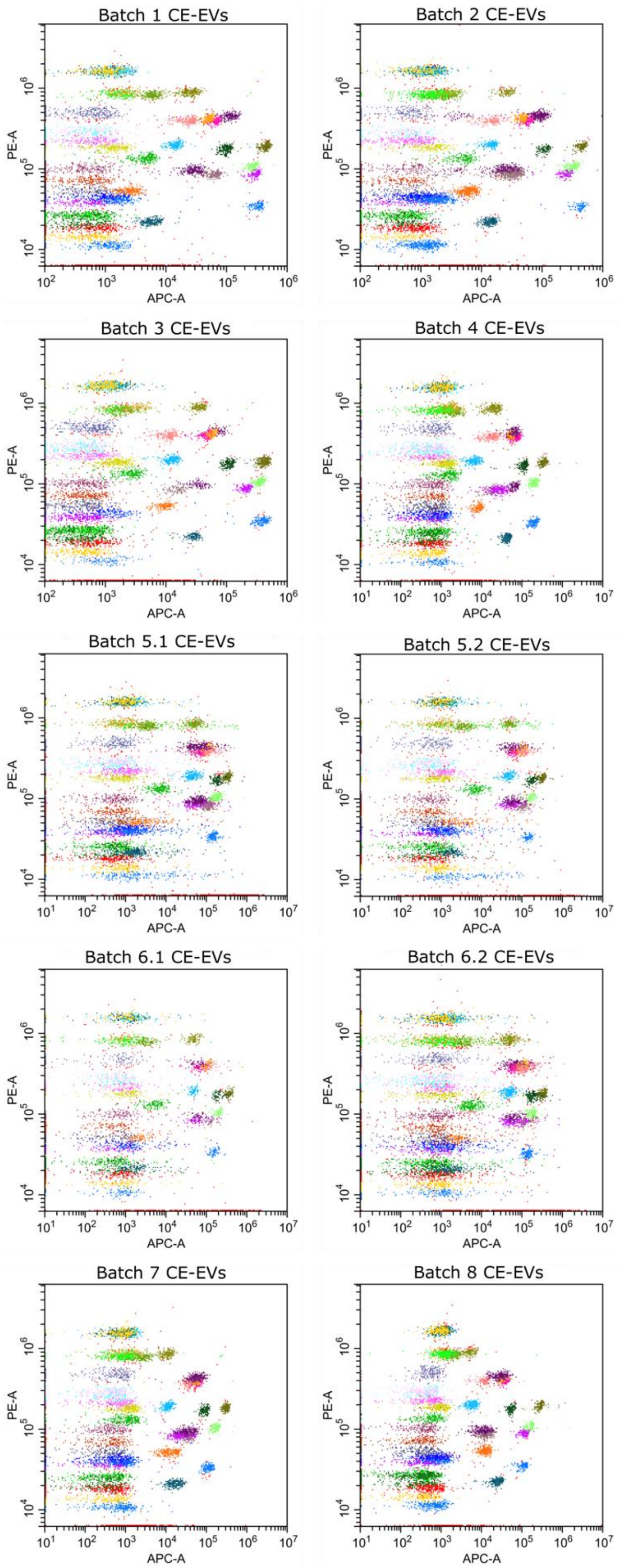

Figure S4. PE (phycoerythrin) vs the detection markers CD9/63/81-Allophycocyanin (APC) showing the APC-signal from each bead type in the P1 area for all batches (n=8).

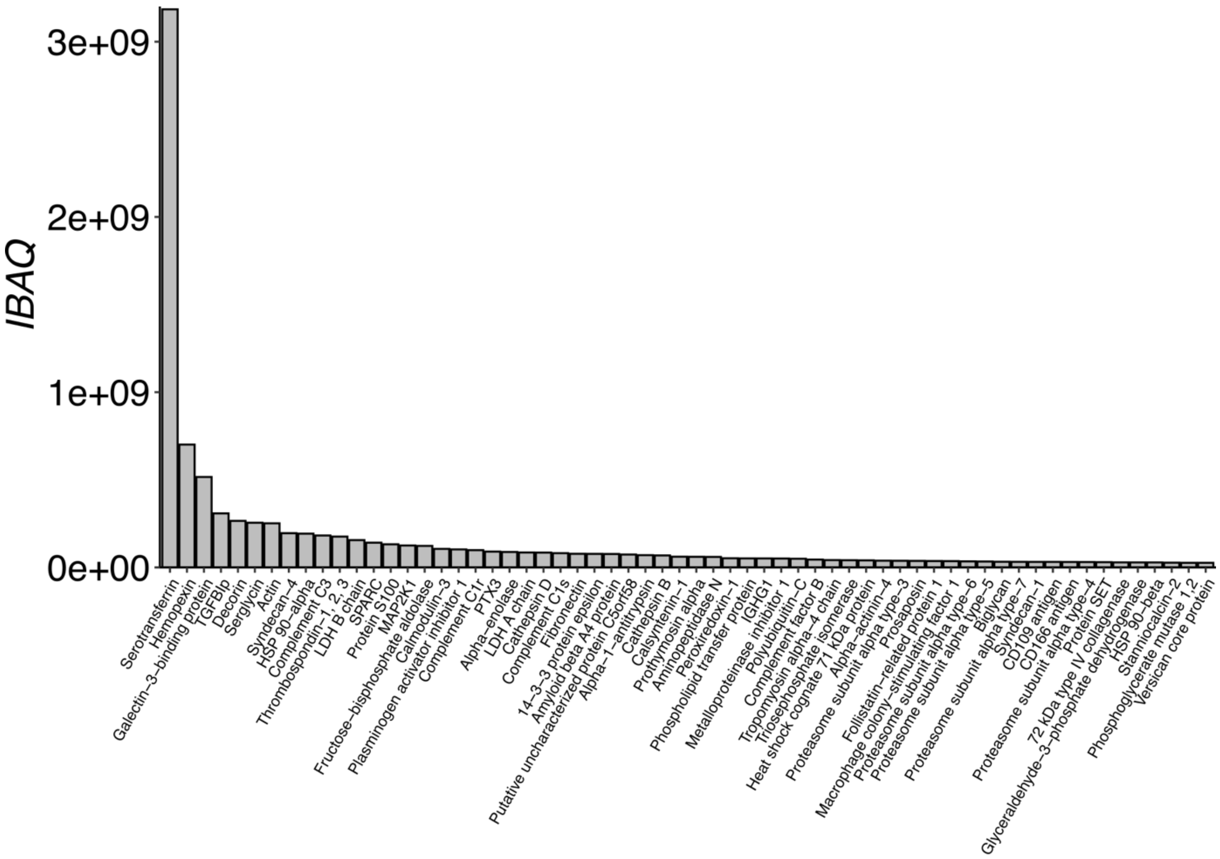

Figure S5. Proteins identified through proteomic analyses with highest IBAQ (intensity based absolute quantification).

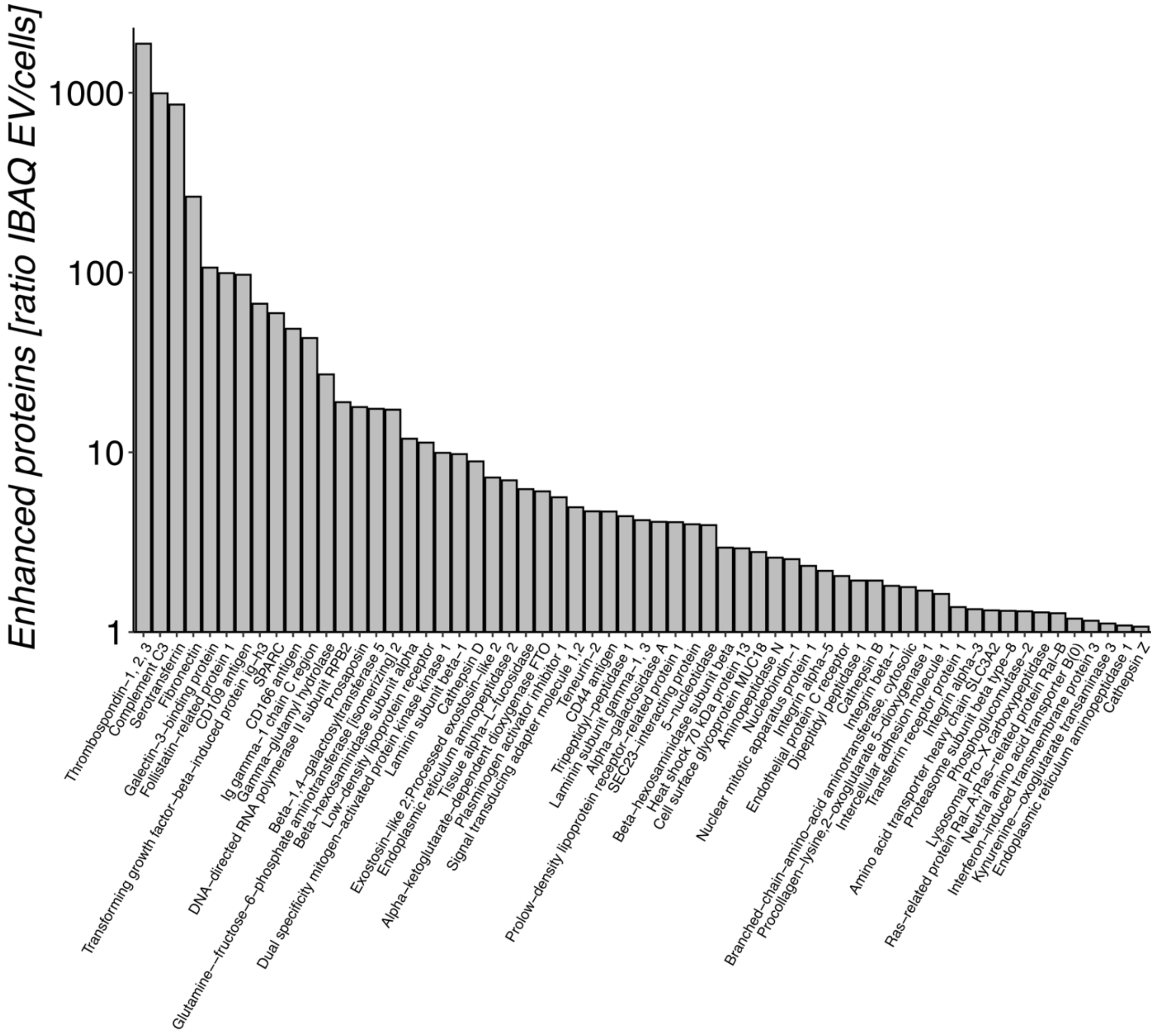

Figure S6. All upregulated proteins in the EVs compared to cells observed in the proteomic analyses. Enhancement is calculated from the IBAQ ratios from EVs compared to cells.

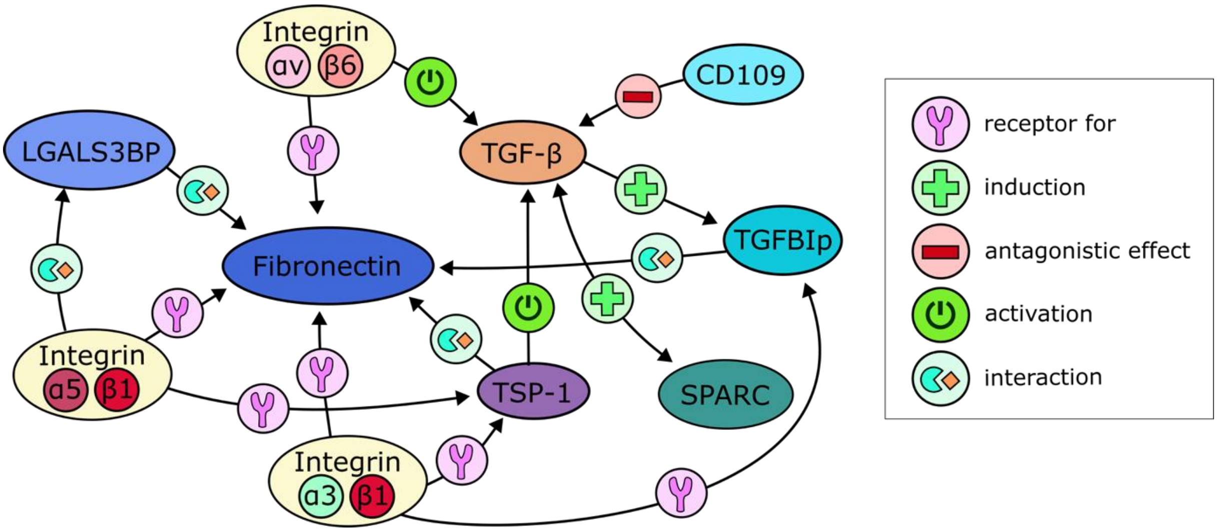

Figure S7. Interaction and signaling of proteins found in the EVs based on the current study and literature.

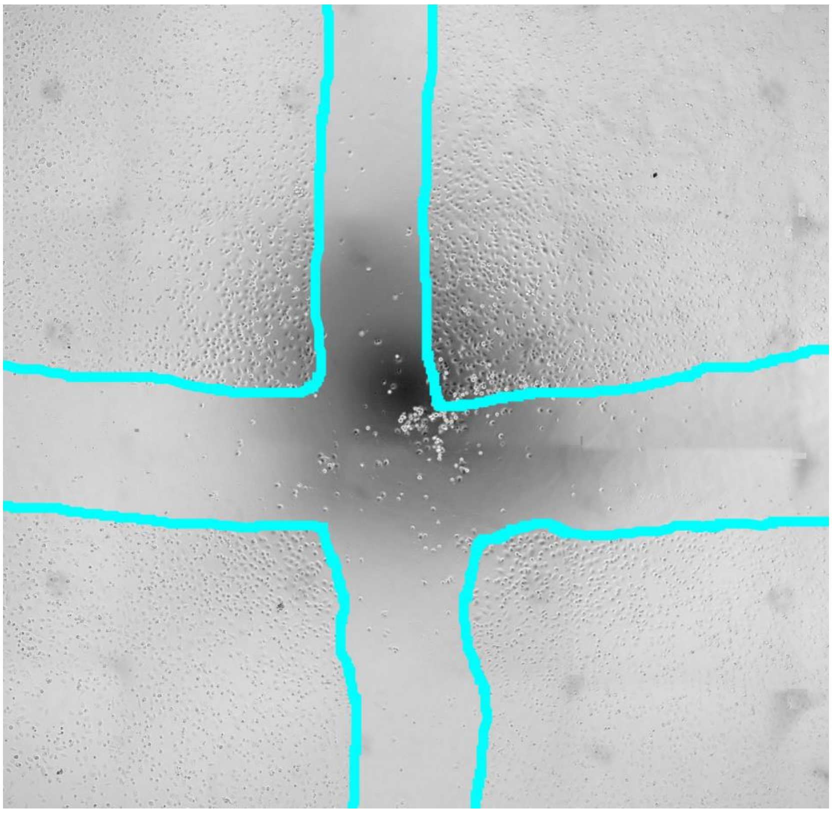

Figure S8. Representative zoomed-in brightfield image showing the scratched region created for the scratch assay. The outlined area (highlighted in blue) demarcates the cell-free zone generated by the scratch, with surrounding corneal epithelial cells.

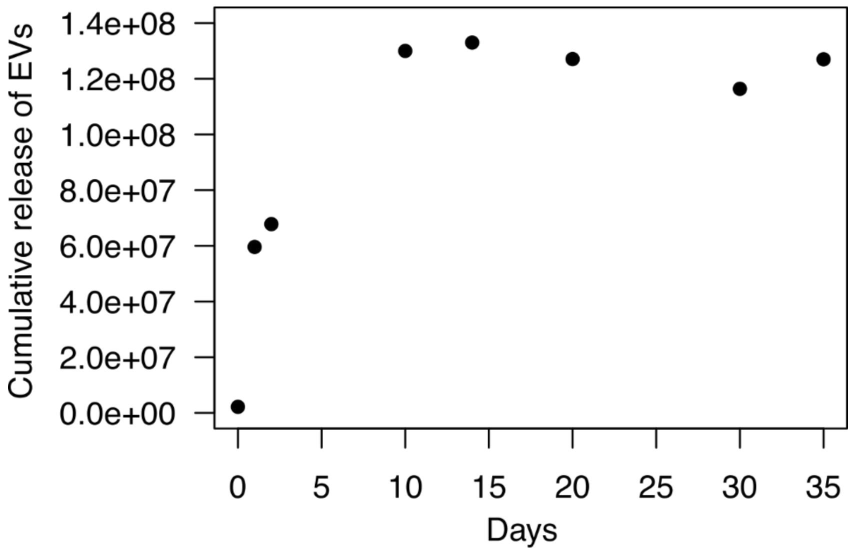

Figure S9. Cumulative release profile of CE-EVs from the hydrogel over a 35-day period.

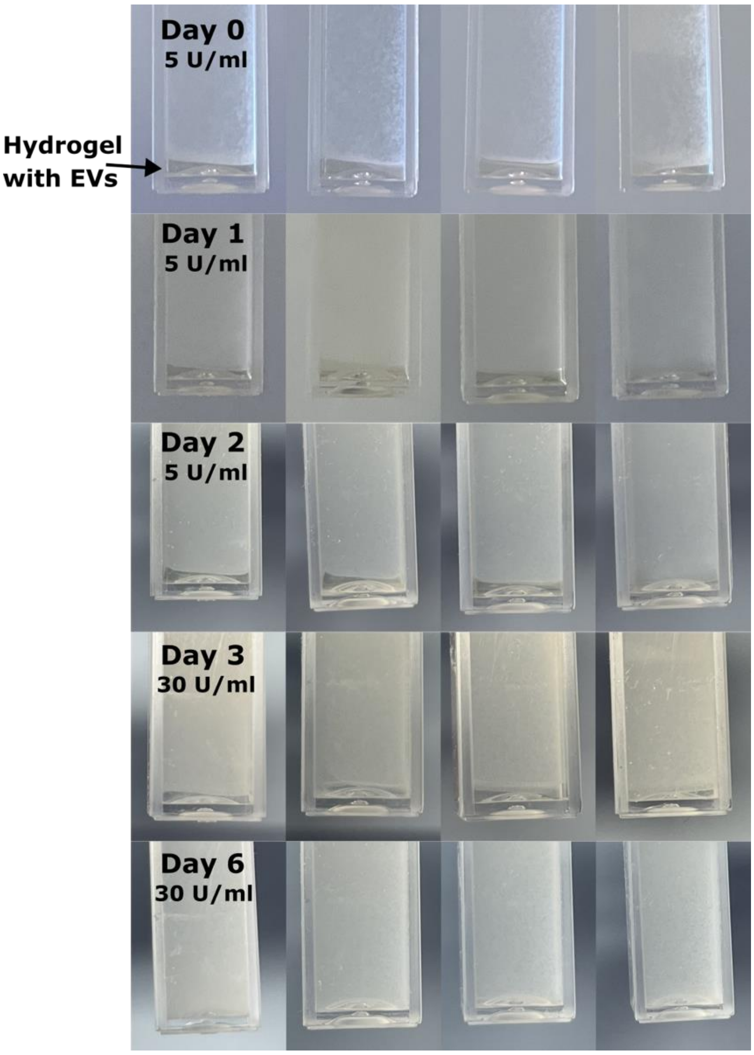

Figure S10. Collagenase degradation of hydrogels containing EVs after release studies. The first 3 days the concentration of collagenase was 5 U/ml and on day 3 it was increased to 30 U/ml.

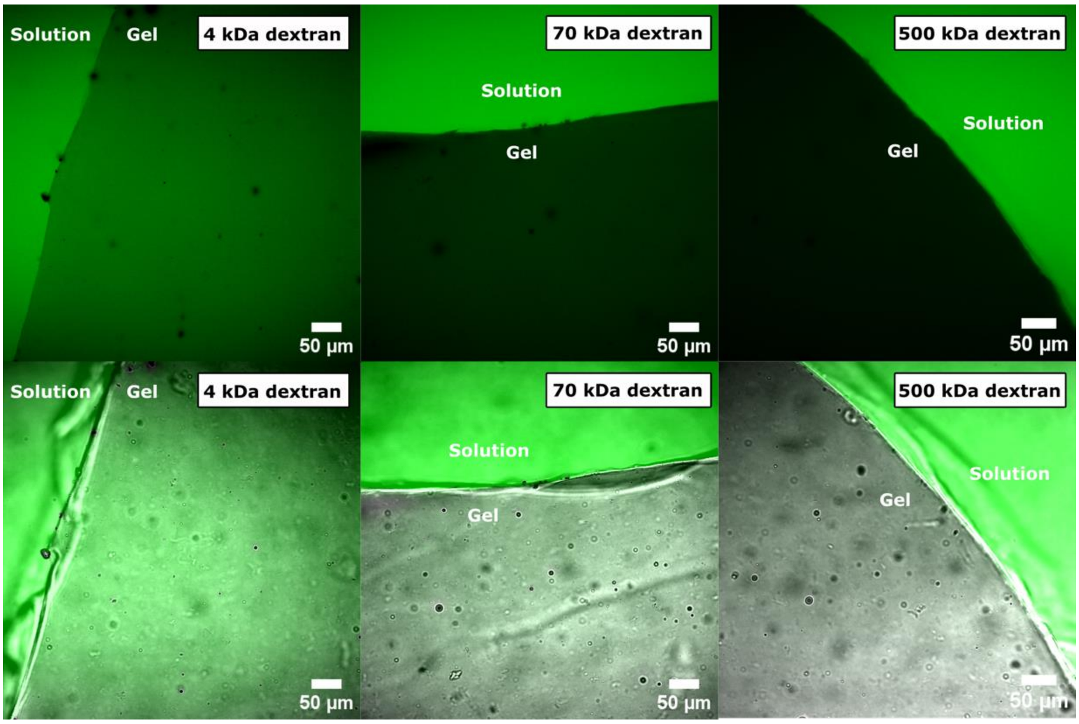

Figure S11. Confocal microscope images of hydrogel probed with various molecular weight FITC-dextran at the interface of solution and hydrogel; fluorescence scan (top) and overlay of brightfield and fluorescence scan (bottom).

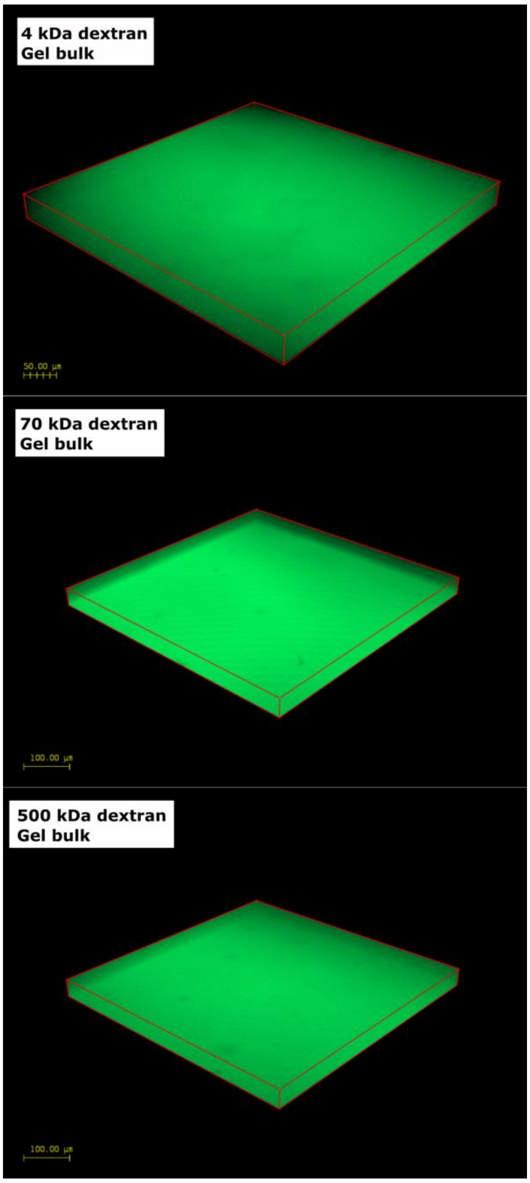

Figure S12. Confocal 3D pictures of hydrogels incubated with different sizes of FITC-dextran's (4k Da, 70 kDa, and 500 kDa). Pictures showing the homogeneity of the bulk phase of the hydrogel (intensities are increased for the higher kDa dextran's to see possible inhomogeneities).

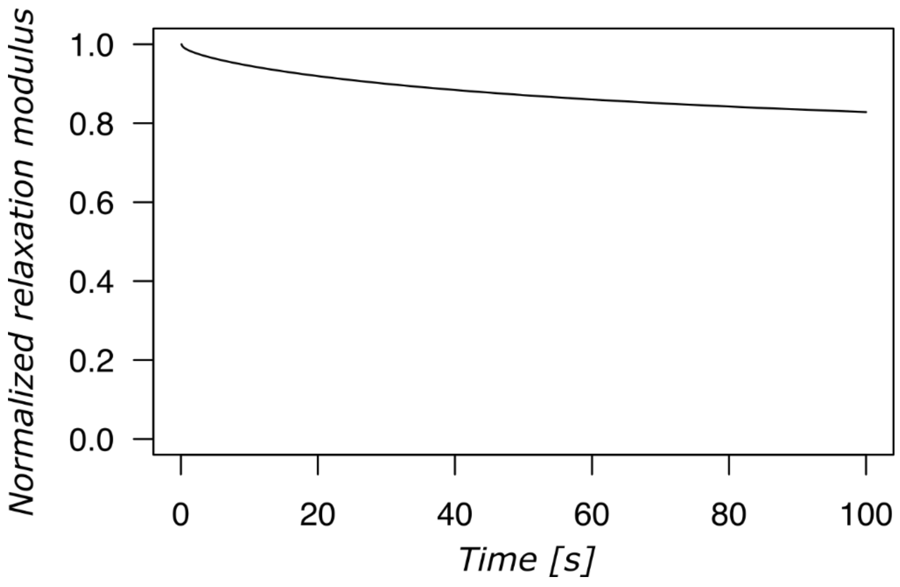

Figure S13. Normalized stress-relaxation of hydrogel.

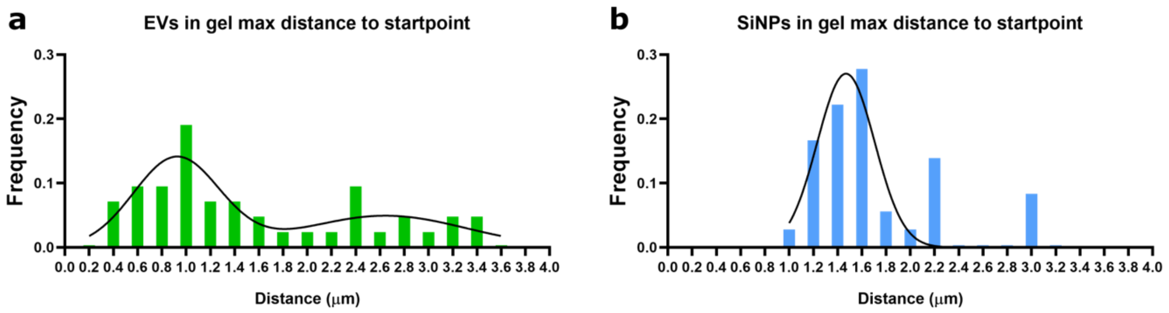

Figure S14. Tracking histograms: a) showing two populations for encapsulated EVs, but b) only one population for silica nanoparticles (SiNPs).

Table S1. List of proteins identified either exclusively or enriched in CE-EVs compared to CE-cells through LC-MS proteomics analyses.

Protein name	Protein IDs (main)	Ratio	Location	Peptides	Nr. of proteins
Pantetheinase	O95497	Only in EVs	Cell membrane	1	5
Oncostatin-M-specific receptor subunit beta	Q99650	Only in EVs	Cell membrane	3	5
Neutral amino acid transporter B(0)	Q15758	1,187914273	Cell membrane	2	5
Neural cell adhesion molecule L1	P32004	Only in EVs	Cell membrane	3	5
Neogenin	Q92859	Only in EVs	Cell membrane	6	5
Nectin-2	Q92692	Only in EVs	Cell membrane	5	5
Natural cytotoxicity triggering receptor 3 ligand 1	Q68D85	Only in EVs	Cell membrane	2	5
Monocarboxylate transporter 4	O15427	0,257210292	Cell membrane	2	6
Melanotransferrin	P08582	Only in EVs	Cell membrane	1	4
Intercellular adhesion molecule 1	P05362	1,629879906	Cell membrane	4	3
Integrin beta-6	P18564	Only in EVs	Cell membrane	1	3
Integrin alpha-V	P06756	Only in EVs	Cell membrane	5	3
Integrin alpha-6	P23229	Only in EVs	Cell membrane	2	3
Integrin alpha-5	P08648	2,191489362	Cell membrane	11	2
Integrin alpha-3	P26006	1,34071461	Cell membrane	8	6
ICOS ligand	O75144	Only in EVs	Cell membrane	5	3
Guanine nucleotide-binding protein G(I)/G(S)/G(O) subunit gamma-12	Q9UBI6	0,191089388	Cell membrane	3	1
Equilibrative nucleobase transporter 1	Q8NBI5	Only in EVs	Cell membrane	1	2
Endothelial protein C receptor	Q9UNN8	2,049964111	Cell membrane	3	2
Discoidin, CUB and LCCL domain-containing protein 2	Q96PD2	Only in EVs	Cell membrane	4	2
Desmoglein-2	Q14126	Only in EVs	Cell membrane	3	2
Chondroitin sulfate proteoglycan 4	Q6UVK1	Only in EVs	Cell membrane	5	2
Cell surface glycoprotein MUC18	P43121	2,788216282	Cell membrane	6	2
CD109 antigen	Q6YHK3	97,20806696	Cell membrane	31	7
Cadherin-2	P19022	Only in EVs	Cell membrane	4	1
Cadherin-13	P55290	Only in EVs	Cell membrane	1	1
C-type mannose receptor 2	Q9UBG0	Only in EVs	Cell membrane	1	1
Brain acid soluble protein 1	P80723	0,438314257	Cell membrane	8	3
AP-2 complex subunit mu	Q96CW1	Only in EVs	Cell membrane	3	1
Aminopeptidase N	P15144	2,593280014	Cell membrane	31	4
Adenylyl cyclase-associated protein 1	Q01518	0,024426777	Cell membrane	22	12
5-nucleotidase	P21589	3,933139628	Cell membrane	4	3
Voltage-dependent calcium channel subunit alpha-2/delta-1	P54289	Only in EVs	Cell membrane	1	5
Tyrosine-protein kinase receptor UFO;Receptor protein-tyrosine kinase	P30530	Only in EVs	Cell membrane	5	3
CD81 antigen	P60033	Only in EVs	Cell membrane	1	8
CD151 antigen	P48509	Only in EVs	Cell membrane	1	4
Teneurin-3	Q9P273	Only in EVs	Cell membrane	4	1
Sushi domain-containing protein 5	O60279	Only in EVs	Cell membrane	3	2

Solute carrier family 2, facilitated glucose transporter member 1	P11166	Only in EVs	Cell membrane	1	1
Sodium/potassium-transporting ATPase subunit beta-3	P54709	0,14035147	Cell membrane	2	4
Sodium/potassium-transporting ATPase subunit alpha-1, 2, 3, 4, Potassium-transporting ATPase alpha chain 1, 2	P05023, P13637, P50993, Q13733, P20648, P54707	0,190432997	Cell membrane	8	20
Semaphorin-7A	O75326	Only in EVs	Cell membrane	2	3
Receptor-type tyrosine-protein phosphatase gamma	P23470	Only in EVs	Cell membrane	2	2
Receptor-type tyrosine-protein phosphatase F	P10586	Only in EVs	Cell membrane	1	4
Inactive tyrosine-protein kinase 7	Q13308	Only in EVs	Cell membrane	1	3
Neuronal cell adhesion molecule	Q92823	Only in EVs	Cell membrane + Extracellular (secreted)	5	5
Nectin-1	Q15223	Only in EVs	Cell membrane + Extracellular (secreted)	4	5
Monocyte differentiation antigen CD14	P08571	Only in EVs	Cell membrane + Extracellular (secreted)	5	5
Macrophage colony-stimulating factor 1	P09603	Only in EVs	Cell membrane + Extracellular (secreted)	1	4
Receptor-type tyrosine-protein phosphatase eta	Q12913	Only in EVs	Cell membrane + Extracellular (secreted)	3	5
Peptidyl-glycine alpha-amidating monooxygenase	P19021	Only in EVs	Cell membrane + Extracellular (secreted) + Cytoplasmic vesicle	5	5
Lactadherin	Q08431	Only in EVs	Cell membrane + Extracellular (secreted) + Cytoplasmic vesicle	2	4
Trans-Golgi network integral membrane protein 2	O43493	Only in EVs	Cell membrane + ER/Golgi	2	7
Teneurin-2	Q9NT68	4,695924132	Cell membrane + ER/Golgi	12	7
Roundabout homolog 1	Q9Y6N7	Only in EVs	Cell membrane + ER/Golgi	1	9
Beta-1,4-galactosyltransferase 1	P15291	Only in EVs	Cell membrane + ER/Golgi + Extracellular (secreted)	25	1
Ras GTPase-activating-like protein IQGAP1	P46940	0,168353829	Cell membrane + Cytoplasm (soluble) + Nucleus	15	5
Annexin A1	P04083	0,007218426	Cell membrane + Cytoplasm (Soluble) + Nucleus + Extracellular (secreted) + Endosome + Cytoplasmic vesicle	27	3
Amyloid-beta precursor protein	P05067	Only in EVs	Cell membrane + Cytoplasm (Soluble) + Nucleus + Extracellular (secreted) + Endosome + Cytoplasmic vesicle + ER/Golgi	7	1
Prolow-density lipoprotein receptor-related protein 1	Q07954	4,088050314	Cell membrane + Cytoplasm (Cytoskeleton/Motor)	7	1
Moesin	P26038	0,177331164	Cell membrane + Cytoplasm (Cytoskeleton/Motor)	31	3
Ezrin	P15311	0,045760445	Cell membrane + Cytoplasm (Cytoskeleton/Motor)	15	1
Vinculin	P18206	0,374310693	Cell membrane + Cytoplasm (Cytoskeleton/Motor)	35	4
Radixin	P35241	0,100558955	Cell membrane + Cytoplasm (Cytoskeleton/Motor)	27	12

Stomatin	P27105	Only in EVs	Cell membrane + Cytoplasm (Cytoskeleton/Motor) + Cytoplasmic vesicles	4	3
Coronin-1C	Q9ULV4	0,12253789	Cell membrane + Cytoplasm (Soluble and Cytoskeleton/Motor) + Endosome	8	12
Guanine nucleotide-binding proteins (G(s), G(i), G(t), G(olf), G(o))	P63092, P63096, A8MTJ3, P11488, P19087, P38405, P09471	0,424818177	Cell membrane + Cytoplasm (Soluble and Cytoskeleton/Motor) + Nucleus	4	35
Guanine nucleotide-binding protein G(I)/G(S)/G(T) subunit beta-2, Guanine nucleotide-binding protein subunit beta-4	P62879, Q9HAV0	0,111826001	Cell membrane + Cytoplasm (Soluble)	8	14
Neuropilin-1	O14786	Only in EVs	Cell membrane + Cytoplasm (Soluble) + Extracellular (Secreted) + Mitochondria	11	5
Nicastrin	Q92542	0,45819563	Cell membrane + Cytoplasmic vesicle	3	13
Integrin beta-1	P05556	1,807707441	Cell membrane + Endosome	13	16
Ephrin type-B receptor 1,2,3,4; Ephrin type-A receptor 3,5,6,7,8; Tyrosine-protein kinase BTK	P54762, P29323, P54753, P54760, P29320, P54756, Q9UF33, Q15375, P29322, Q06187	Only in EVs	Cell membrane + Endosome + Cytoplasm (soluble) + Nucleus	2	38
Glypican-1	P35052	Only in EVs	Cell membrane + Endosome + Extracellular (Secreted)	3	3
Interferon-induced transmembrane protein 3	Q01628	1,156185427	Cell membrane + Endosome + Lysosome + Cytoplasm (soluble)	1	7
CD63 antigen	P08962	Only in EVs	Cell membrane + Endosome + Lysosome + Extracellular (Secreted)	1	7
HLA-A, HLA-C	Q53Z42, P10321	0,421065356	Cell membrane + ER/Golgi	9	34
Bone marrow stromal antigen 2	Q10589	0,324187707	Cell membrane + ER/Golgi + Cytoplasm (soluble) + Endosome	1	2
Basigin 1, 2, 3, 4	P35613	0,075977595	Cell membrane + ER/Golgi + Endosome	2	11
Mesothelin	Q13421	Only in EVs	Cell membrane + ER/Golgi + Extracellular (Secreted)	1	4
Syntenin-1	O00560	0,549553737	Cell membrane + ER/Golgi + Extracellular (Secreted) + Cytosol (Soluble and cytoskeleton/motor) + Nucleus	2	5
Low-density lipoprotein receptor	P01130	11,32634055	Cell membrane + ER/Golgi + Lysosome + Endosome	8	10
Calsyntenin-1	O94985	Only in EVs	Cell membrane + ER/Golgi + Nucleus	1	1
Protein CutA	O60888	0,640372443	Cell membrane + Extracellular (Secreted)	3	5
Leucyl-cystinyl aminopeptidase	Q9UIQ6	Only in EVs	Cell membrane + Extracellular (Secreted)	5	4
Interleukin-6 receptor subunit beta	P40189	Only in EVs	Cell membrane + Extracellular (Secreted)	1	4
Dipeptidyl peptidase 4	P27487	Only in EVs	Cell membrane + Extracellular (Secreted)	10	2

CD59 glycoprotein	P13987	0,548688845	Cell membrane + Extracellular (Secreted)	1	4
CD44 antigen	P16070	4,684431535	Cell membrane + Extracellular (Secreted)	5	28
CD166 antigen	Q13740, Q13740-3	48,68090057	Cell membrane + Extracellular (secreted)	17	6
Attractin	O75882	Only in EVs	Cell membrane + Extracellular (Secreted)	1	1
Transferrin receptor protein 1	P02786	1,377005611	Cell membrane + Extracellular (Secreted)	19	11
Syndecan-4	P31431-2, P31431	Only in EVs	Cell membrane + Extracellular (Secreted)	3	2
Syndecan-1	P18827	Only in EVs	Cell membrane + Extracellular (secreted)	1	3
Ceroid-lipofuscinosis neuronal protein 5	O75503	Only in EVs	Cell membrane + Lysosome	1	2
Platelet-derived growth factor receptor beta	P09619	0,022043487	Cell membrane + Lysosome + Cytoplasmic vesicle	2	4
Amino acid transporter heavy chain SLC3A2	P08195	1,319813991	Cell membrane + Lysosome + Nucleus	7	23
Choline transporter-like protein 1	Q8WWI5	Only in EVs	Cell membrane + Mitochondia	1	2
Ras-related protein Ral-A;Ras-related protein Ral-B	P11233, P11234	1,273228789	Cell membrane + Mitochondia	3	10
Protocadherin Fat 1	Q14517	Only in EVs	Cell membrane + Nucleus	1	7
Amyloid beta precursor like protein 2	Q06481	Only in EVs	Cell membrane + Nucleus	8	1
HLA-B	P01889	0,843175983	Cell membrane+ ER/Golgi	7	15
Profilin-1	P07737	0,016011963	Cytoplasm (Cytoskeleton/Motor)	12	4
Myosin light polypeptide 6, Myosin light chain 6B	P60660, P14649	0,007383658	Cytoplasm (Cytoskeleton/Motor)	7	16
Myosin light chain 1/3, Myosin light chain 3	P05976, P08590	0,022151635	Cytoplasm (Cytoskeleton/Motor)	1	3
Filamin-B	O75369	0,328229256	Cytoplasm (Cytoskeleton/Motor)	85	15
Filamin-A	P21333	0,153744716	Cytoplasm (Cytoskeleton/Motor)	73	10
F-actin-capping protein subunit beta	P47756	0,020446103	Cytoplasm (Cytoskeleton/Motor)	9	7
F-actin-capping protein subunit alpha-1	P52907	0,020088827	Cytoplasm (Cytoskeleton/Motor)	9	1
Dual specificity mitogen-activated protein kinase kinase 1	Q02750	9,927697442	Cytoplasm (Cytoskeleton/Motor)	4	12
Cofilin-1	P23528	0,008752741	Cytoplasm (Cytoskeleton/Motor)	14	7
Calmodulin-1,2,3	P0DP25, P0DP24, P0DP23	0,248314897	Cytoplasm (Cytoskeleton/Motor)	8	15
Alpha-centractin	P61163	0,058680618	Cytoplasm (Cytoskeleton/Motor)	4	3
Actin, alpha skeletal muscle, Actin, alpha cardiac muscle 1, Actin, gamma-enteric smooth muscle, Actin, aortic smooth muscle	P68133, P68032, P63267, P62736	0,065723024	Cytoplasm (Cytoskeleton/Motor)	22	11
Tubulin beta-4B chain	P68371, (P04350)	0,006900402	Cytoplasm (Cytoskeleton/Motor)	26	9
Tubulin beta chain	P07437, Q9H4B7	0,008826327	Cytoplasm (Cytoskeleton/Motor)	23	10
Tubulin alpha-1B chain	P68363	0,02433094	Cytoplasm (Cytoskeleton/Motor)	27	3
Tropomyosin alpha-4 chain, Coiled-coil domain-containing protein 57	P67936, Q2TAC2	0,183967284	Cytoplasm (Cytoskeleton/Motor)	24	20
Tropomyosin alpha-3 chain	P06753	0,113113695	Cytoplasm (Cytoskeleton/Motor)	22	7
Tropomyosin alpha-1 chain	P09493	0,011928604	Cytoplasm (Cytoskeleton/Motor)	18	14
Thymosin beta-10	P63313	0,004589248	Cytoplasm (Cytoskeleton/Motor)	3	2
Talin-1	Q9Y490, Q9Y4G6	0,229713754	Cytoplasm (Cytoskeleton/Motor)	39	4
Spectrin alpha chain, non-erythrocytic 1	Q13813	0,315559757	Cytoplasm (Cytoskeleton/Motor)	26	7

Plectin, Epiplakin, Microtubule-actin cross-linking factor 1	Q15149, P58107, Q9UPN3	0,095338397	Cytoplasm (Cytoskeleton/Motor) + Cytoplasm (Soluble) + ER/Golgi	93	22
Nuclear migration protein nudC	Q9Y266	0,008364645	Cytoplasm (Cytoskeleton/Motor) + Nucleus	11	3
Actin, cytoplasmic 1	P60709	0,080029247	Cytoplasm (Cytoskeleton/Motor) + Nucleus	35	20
Actin-related protein 2	P61160	0,019606725	Cytoplasm (Cytoskeleton/Motor) + Nucleus	9	4
Actin-related protein 2/3 complex subunit 3	O15145	0,186689602	Cytoplasm (Cytoskeleton/Motor) + Nucleus	3	3
Actin-related protein 2/3 complex subunit 4	P59998	0,078705595	Cytoplasm (Cytoskeleton/Motor) + Nucleus	5	11
Nuclear mitotic apparatus protein 1	Q14980	2,335186104	Cytoplasm (Cytoskeleton/Motor) + Nucleus + Cell membrane	3	8
Microtubule-associated protein 1 A, B	P46821, P78559	0,046498001	Cytoplasm (Soluble and Cytoskeleton/Motor)	7	8
Katanin p60 ATPase-containing subunit A-like 2	Q8IYT4	Only in EVs	Cytoplasm (Soluble and Cytoskeleton/Motor)	4	4
Fructose-bisphosphate aldolase A, B	P04075, P05062	0,4775852	Cytoplasm (Soluble and Cytoskeleton/Motor)	32	12
Filamin-C	Q14315	0,311861577	Cytoplasm (Soluble and Cytoskeleton/Motor)	35	2
Fascin	Q16658	0,015795942	Cytoplasm (Soluble and Cytoskeleton/Motor)	14	4
Dihydropyrimidinase-related protein 1, 2	Q16555, Q14194	0,053461017	Cytoplasm (Soluble and Cytoskeleton/Motor)	14	8
WD repeat-containing protein 1	O75083	0,030566921	Cytoplasm (Soluble and Cytoskeleton/Motor)	15	10
T-complex protein 1 subunit theta	P50990	0,068356721	Cytoplasm (Soluble and Cytoskeleton/Motor)	30	5
T-complex protein 1 subunit epsilon	P48643	0,045096996	Cytoplasm (Soluble and Cytoskeleton/Motor)	18	7
T-complex protein 1 subunit delta	P50991	0,049727194	Cytoplasm (Soluble and Cytoskeleton/Motor)	18	2
T-complex protein 1 subunit alpha	P17987	0,081642448	Cytoplasm (Soluble and Cytoskeleton/Motor)	16	11
Plastin-1, 2, 3	P13797, P13796, Q14651	0,192967706	Cytoplasm (Soluble and Cytoskeleton/Motor) + Cell membrane	13	10
Alpha-actinin-1, 2, 3	P12814, P35609, Q08043	0,545495495	Cytoplasm (Soluble and Cytoskeleton/Motor) + Cell membrane	48	38
Spectrin beta chain, non-erythrocytic 1	Q01082	0,075843719	Cytoplasm (Soluble and Cytoskeleton/Motor) + Cell membrane	17	5
Rho guanine nucleotide exchange factor 18	Q6ZSZ5	0,729830187	Cytoplasm (Soluble and Cytoskeleton/Motor) + Cell membrane	1	10
Vimentin, Desmin, Glial fibrillary acidic protein, Neurofilament medium polypeptide, Neurofilament light polypeptide, Neurofilament heavy polypeptide	P08670, P17661, P14136, P07197, Q8N1N4, P07196, P12036	0,014931794	Cytoplasm (Soluble and Cytoskeleton/Motor) + Cell membrane + Nucleus + Extracellular (Secreted)	64	30
Myosin-9, Myosin 14	P35579, Q7Z406	0,042624884	Cytoplasm (Soluble and Cytoskeleton/Motor) + Cytoplasmic vesicle	81	19
Programmed cell death 6-interacting protein	Q8WUM4	0,164764107	Cytoplasm (Soluble and Cytoskeleton/Motor) + Extracellular (secreted)	11	8
Fructose-bisphosphate aldolase C;Fructose-bisphosphate aldolase	P09972	0,086193716	Cytoplasm (Soluble and Cytoskeleton/Motor) + Extracellular (secreted)	6	6

Transgelin-2	P37802	0,012439743	Cytoplasm (Soluble and Cytoskeleton/Motor) + Extracellular (Secreted)	17	4
Glyceraldehyde-3-phosphate dehydrogenase	P04406, O14556	0,035194316	Cytoplasm (Soluble and Cytoskeleton/Motor) + Nucleus	29	4
Coactosin-like protein	Q14019	0,053085296	Cytoplasm (Soluble and Cytoskeleton/Motor) + Nucleus	5	2
Alpha-actinin-4, Spectrin beta chain, non-erythrocytic 4	O43707, Q9H254	0,40596408	Cytoplasm (Soluble and Cytoskeleton/Motor) + Nucleus	53	11
Serine/threonine-protein phosphatase 2A catalytic subunit alpha isoform, beta isoform	P67775, P62714	0,039875644	Cytoplasm (Soluble and Cytoskeleton/Motor) + Nucleus	7	13
Heat shock 70 kDa protein 1A, B	P0DMV9, P0DMV8	0,164669098	Cytoplasm (Soluble and Cytoskeleton/Motor) + Nucleus + Extracellular (secreted)	29	5
Peptidyl-prolyl cis-trans isomerase FKBP4	Q02790	0,109408696	Cytoplasm (Soluble and Cytoskeleton/Motor) + Nucleus + Mitochondria	22	4
Small ribosomal subunit protein uS3	P23396	0,006320313	Cytoplasm (Soluble and Cytoskeleton/Motor) + Nucleus + Mitochondria	10	14
Putative heat shock protein HSP 90-beta 4	Q58FF6	0,357121401	Cytoplasm (Soluble)	6	1
Putative heat shock protein HSP 90-beta 2	Q58FF8	0,614658635	Cytoplasm (Soluble)	10	1
Purine nucleoside phosphorylase	P00491	0,111103963	Cytoplasm (Soluble)	11	5
Protein phosphatase 1G	O15355	0,074627604	Cytoplasm (Soluble)	6	1
Protein Niban 2	Q96TA1	0,049866536	Cytoplasm (Soluble)	11	2
Phosphoserine aminotransferase	Q9Y617	0,073321404	Cytoplasm (Soluble)	4	2
Phosphoribosylformylglycinamidine synthase	O15067	0,068047132	Cytoplasm (Soluble)	10	4
Phosphoglycerate kinase 1, 2	P00558, P07205	0,083432461	Cytoplasm (Soluble)	23	3
Phosphoglucomutase-2	Q96G03	1,305293703	Cytoplasm (Soluble)	4	5
Phosphoglucomutase-1	P36871	0,073717642	Cytoplasm (Soluble)	7	4
Phosphoacetylglucosamine mutase	O95394	0,059968344	Cytoplasm (Soluble)	7	24
Peroxiredoxin-2	P32119	0,125954804	Cytoplasm (Soluble)	12	3
Peroxiredoxin-1	Q06830	0,109402157	Cytoplasm (Soluble)	15	3
Nicotinamide N-methyltransferase	P40261	0,007340663	Cytoplasm (Soluble)	9	1
NADP-dependent malic enzyme	P48163	0,499003503	Cytoplasm (Soluble)	2	2
Malate dehydrogenase, cytoplasmic	P40925	0,233069779	Cytoplasm (Soluble)	12	9
Large ribosomal subunit protein P2	P05387	0,023143152	Cytoplasm (Soluble)	7	2
L-lactate dehydrogenase B chain;L-lactate dehydrogenase	P07195	0,480743733	Cytoplasm (Soluble)	15	6
Kynureninase	Q16719	0,432568188	Cytoplasm (Soluble)	8	6
Isocitrate dehydrogenase [NADP]	O75874	Only in EVs	Cytoplasm (Soluble)	3	4
Hypoxanthine-guanine phosphoribosyltransferase	P00492	0,164270594	Cytoplasm (Soluble)	7	1
Hsp90 co-chaperone Cdc37	Q16543	0,040338405	Cytoplasm (Soluble)	9	5
Hsc70-interacting protein	P50502	0,060426459	Cytoplasm (Soluble)	8	6
Heat shock 70 kDa protein 4	P34932	0,276889429	Cytoplasm (Soluble)	23	2
Glutathione synthetase	P48637	0,643042904	Cytoplasm (Soluble)	17	14
Glutathione S-transferase omega-1	P78417	0,075760582	Cytoplasm (Soluble)	14	5

Glutamine--fructose-6-phosphate aminotransferase [isomerizing] 2	O94808	17,2572632	Cytoplasm (Soluble)	3	2
Glucosamine-6-phosphate isomerase 2	Q8TDQ7	0,510837948	Cytoplasm (Soluble)	3	5
Glucosamine-6-phosphate isomerase 1	P46926	0,43916926	Cytoplasm (Soluble)	4	7
Fumarylacetoacetase	P16930	0,052123101	Cytoplasm (Soluble)	7	6
Ferritin light chain	P02792	Only in EVs	Cytoplasm (Soluble)	1	3
Ferritin heavy chain	P02794	Only in EVs	Cytoplasm (Soluble)	6	2
Fatty acid synthase	P49327	0,340840629	Cytoplasm (Soluble)	12	4
Farnesyl pyrophosphate synthase	P14324	0,342914296	Cytoplasm (Soluble)	6	6
Eukaryotic translation initiation factor 3 subunit M	Q7L2H7	0,048884242	Cytoplasm (Soluble)	7	5
Eukaryotic translation initiation factor 3 subunit F	O00303	0,026996096	Cytoplasm (Soluble)	7	5
Eukaryotic translation initiation factor 3 subunit C	Q99613	0,035978651	Cytoplasm (Soluble)	6	4
Eukaryotic translation initiation factor 3 subunit B	P55884	0,131077168	Cytoplasm (Soluble)	14	4
Eukaryotic translation initiation factor 3 subunit A	Q14152	0,010634302	Cytoplasm (Soluble)	18	2
Eukaryotic translation initiation factor 2 subunit 2	P20042	0,358012319	Cytoplasm (Soluble)	6	1
Eukaryotic translation initiation factor 2 subunit 1	P05198	0,010809311	Cytoplasm (Soluble)	12	3
Eukaryotic initiation factor 4A-I	P60842	0,011563747	Cytoplasm (Soluble)	24	20
Elongation factor 1-beta	P24534	0,042852633	Cytoplasm (Soluble)	4	4
Dipeptidyl peptidase 3	Q9NY33	0,058962585	Cytoplasm (Soluble)	7	8
Deoxyhypusine synthase	P49366	0,066623571	Cytoplasm (Soluble)	4	9
Cytosolic non-specific dipeptidase	Q96KP4	0,06450992	Cytoplasm (Soluble)	11	16
Cytosol aminopeptidase	P28838	0,210717048	Cytoplasm (Soluble)	16	3
Cytoplasmic aconitate hydratase	P21399	0,087083601	Cytoplasm (Soluble)	13	1
Cysteine dioxygenase type 1	Q16878	0,169393489	Cytoplasm (Soluble)	1	1
Cullin-associated NEDD8-dissociated protein 1, 2	Q86VP6, O75155	0,50598859	Cytoplasm (Soluble)	16	10
Caspase recruitment domain-containing protein 10	Q9BWT7	Only in EVs	Cytoplasm (Soluble)	11	1
Caprin-1	Q14444	0,061130283	Cytoplasm (Soluble)	13	4
Branched-chain-amino-acid aminotransferase, cytosolic	P54687	1,779709025	Cytoplasm (Soluble)	2	6
Bifunctional purine biosynthesis protein ATIC	P31939	0,016451629	Cytoplasm (Soluble)	16	5
Bifunctional phosphoribosylaminoimidazole carboxylase/phosphoribosylaminoimidazole succinocarboxamide synthetase	P22234	0,063484934	Cytoplasm (Soluble)	11	4
ATP-citrate synthase	P53396	0,085066251	Cytoplasm (Soluble)	23	4
Aspartate aminotransferase	P17174	0,389470182	Cytoplasm (Soluble)	9	2
Annexin A6	P08133	0,007284919	Cytoplasm (Soluble)	28	12
Aminoacylase-1	Q03154	0,067691977	Cytoplasm (Soluble)	2	9
Acylamino-acid-releasing enzyme	P13798	0,97807363	Cytoplasm (Soluble)	6	6
6-phosphogluconate dehydrogenase, decarboxylating	P52209	0,307589257	Cytoplasm (Soluble)	5	6
14-3-3 protein zeta/delta	P63104	0,076610388	Cytoplasm (Soluble)	17	11
14-3-3 protein theta	P27348	0,021256114	Cytoplasm (Soluble)	10	2
14-3-3 protein gamma	P61981	0,150533712	Cytoplasm (Soluble)	13	1
14-3-3 protein beta/alpha	P31946	0,012270304	Cytoplasm (Soluble)	13	6

1,4-alpha-glucan-branching enzyme	Q04446	0,051326366	Cytoplasm (Soluble)	6	2
UTP--glucose-1-phosphate uridylyltransferase	Q16851-2	0,050392747	Cytoplasm (Soluble)	18	17
Ubiquitin thioesterase OTUB1	Q96FW1	0,052409513	Cytoplasm (Soluble)	6	6
Tryptophan--tRNA ligase	P23381	0,103202027	Cytoplasm (Soluble)	13	22
Triosephosphate isomerase	P60174	0,105142638	Cytoplasm (Soluble)	17	6
Thymidine phosphorylase	P19971	0,03183394	Cytoplasm (Soluble)	12	8
Threonine--tRNA ligase	P26639	0,220498383	Cytoplasm (Soluble)	11	9
Thimet oligopeptidase	P52888	Only in EVs	Cytoplasm (Soluble)	2	7
T-complex protein 1 subunit zeta 1,2	P40227, Q92526	0,079243163	Cytoplasm (Soluble)	18	6
T-complex protein 1 subunit gamma	P49368	0,057705123	Cytoplasm (Soluble)	17	9
T-complex protein 1 subunit beta	P78371	0,021170446	Cytoplasm (Soluble)	20	4
Serpin B6, B8	P35237, P50452-2	0,076879575	Cytoplasm (Soluble)	11	12
Secernin-1	Q12765	0,093356853	Cytoplasm (Soluble)	5	6
S-adenosylmethionine synthase isoform type-2	P31153, Q00266	0,149823433	Cytoplasm (Soluble)	6	3
Ribonuclease inhibitor	P13489	0,091087175	Cytoplasm (Soluble)	4	7
Rho GDP-dissociation inhibitor 1	P52565	0,0437428	Cytoplasm (Soluble)	6	7
Prolyl endopeptidase	P48147	0,335741432	Cytoplasm (Soluble)	3	2
Leukotriene A-4 hydrolase	P09960	0,104018935	Cytoplasm (Soluble)	7	4
L-lactate dehydrogenase A chain	P00338	0,221698874	Cytoplasm (Soluble)	20	21
Glycogen phosphorylase (liver and muscle form)	P06737, P11217	0,138920418	Cytoplasm (Soluble)	6	5
Eukaryotic translation initiation factor 3 subunit L	Q9Y262	0,112574095	Cytoplasm (Soluble)	4	4
Arginine--tRNA ligase, cytoplasmic	P54136	0,097611416	Cytoplasm (Soluble)	10	4
6-phosphogluconolactonase	O95336	0,087770368	Cytoplasm (Soluble)	2	3
Calpain-2 catalytic subunit	P17655	0,129274668	Cytoplasm (Soluble) + Cell membrane	10	2
Calpain small subunit 1, 2	P04632, Q96L46	0,041326482	Cytoplasm (Soluble) + Cell membrane	7	10
Alpha-enolase	P06733	0,089504602	Cytoplasm (Soluble) + Cell membrane	34	16
Aldo-keto reductase family 1 member A1	P14550	0,07207956	Cytoplasm (Soluble) + Cell membrane	6	4
Ubiquitin carboxyl-terminal hydrolase 14	P54578	0,050178422	Cytoplasm (Soluble) + Cell membrane	11	5
Ras-related protein Rab-1B	Q9H0U4	0,047959824	Cytoplasm (Soluble) + Cell membrane	6	3
Rab GDP dissociation inhibitor beta	P50395	0,072905675	Cytoplasm (Soluble) + Cell membrane	15	8
Guanine nucleotide-binding protein G(I)/G(S)/G(T) subunit beta-1, 3	P62873, P16520	0,202576336	Cytoplasm (Soluble) + Cell membrane + Extracellular (secreted)	6	12
Lactoylglutathione lyase	Q04760	0,048701191	Cytoplasm (Soluble) + Cell membrane + Nucleus	8	2
Bleomycin hydrolase	Q13867	0,09553761	Cytoplasm (Soluble) + Cytoplasmic vesicle	3	6
Vacuolar protein sorting-associated protein 35	Q96QK1	0,094683007	Cytoplasm (soluble) + Endosome	5	1
Signal transducing adapter molecule 1,2	Q92783, O75886	4,94541346	Cytoplasm (Soluble) + Endosome	3	4
Peroxiredoxin-4	Q13162	0,060345034	Cytoplasm (Soluble) + ER/Golgi	9	4
Ubiquitin carboxyl-terminal hydrolase isozyme L1	P09936	0,097137944	Cytoplasm (Soluble) + ER/Golgi	9	5
60S ribosomal protein L18	Q07020	0,016124398	Cytoplasm (Soluble) + ER/Golgi	2	8

Coatomer subunit epsilon	O14579	0,029254415	Cytoplasm (Soluble) + ER/Golgi + Cytoplasmic vesicle	11	5
Protein SET, Protein SETSIP	Q01105, P0DME0	0,839168922	Cytoplasm (Soluble) + ER/Golgi + Nucleus	9	8
Interferon-induced GTP-binding protein Mx1, 2	P20591, P20592-2	0,016703951	Cytoplasm (Soluble) + ER/Golgi + Nucleus	17	14
Protein S100-A13	Q99584	0,018238361	Cytoplasm (Soluble) + Extracellular (secreted)	5	1
Plasminogen activator inhibitor 2	P05120	0,257650442	Cytoplasm (Soluble) + Extracellular (secreted)	11	6
Annexin A5	P08758	0,015803781	Cytoplasm (Soluble) + Extracellular (secreted)	17	5
Ubiquitin-like protein ISG15	P05161	0,09299442	Cytoplasm (Soluble) + Extracellular (secreted)	5	3
Phosphoglycerate mutase 1, 2	P18669, P15259	0,21016211	Cytoplasm (Soluble) + Extracellular (secreted) + Nucleus	12	3
26S proteasome non-ATPase regulatory subunit 6	Q15008	0,029026946	Cytoplasm (Soluble) + Extracellular (secreted) + Nucleus	11	7
26S proteasome non-ATPase regulatory subunit 3	O43242	0,204997173	Cytoplasm (Soluble) + Extracellular (secreted) + Nucleus	4	2
26S proteasome non-ATPase regulatory subunit 2	Q13200	0,016929025	Cytoplasm (Soluble) + Extracellular (secreted) + Nucleus	18	7
26S proteasome non-ATPase regulatory subunit 12	O00232	0,033962624	Cytoplasm (Soluble) + Extracellular (secreted) + Nucleus	4	2
Clusterin	P10909	Only in EVs	Cytoplasm (Soluble) + Extracellular (Secreted) + ER/Golgi + Mitochondia + Cytoplasmic vesicle + Nucleus	3	2
Proprotein convertase subtilisin/kexin type 9	Q8NBP7	Only in EVs	Cytoplasm (soluble) + Extracellular (secreted) + Lysosome + Endosome + ER/Golgi	2	6
Glycine--tRNA ligase	P41250	0,035683242	Cytoplasm (Soluble) + Extracellular (secreted) + Mitochondria	14	16
Glucose-6-phosphate isomerase, Homeobox protein SIX5	P06744, Q8N196	0,20690998	Cytoplasm (Soluble) + Extracellular (secreted) + Nucleus	20	22
Synaptic vesicle membrane protein VAT-1 homolog	Q99536	0,062095436	Cytoplasm (Soluble) + Mitochodria	3	8
Kynurenine--oxoglutarate transaminase 3	Q6YP21	1,116253823	Cytoplasm (Soluble) + Mitochondia	2	4
Interferon-induced protein with tetratricopeptide repeats 3	O14879	0,037942371	Cytoplasm (Soluble) + Mitochondia	5	3
Histidine--tRNA ligase, cytoplasmic and mitochondrial	P12081, P49590	0,04235918	Cytoplasm (Soluble) + Mitochondia	10	28
Glutamate--cysteine ligase catalytic subunit	P48506	0,475292689	Cytoplasm (Soluble) + Mitochondia	1	5
Puromycin-sensitive aminopeptidase	P55786	0,272618102	Cytoplasm (Soluble) + Nucleus	13	15
Protein S100-A11	P31949	0,008765368	Cytoplasm (Soluble) + Nucleus	5	1
Proteasome subunit beta type-9	P28065	0,442213623	Cytoplasm (Soluble) + Nucleus	5	8
Proteasome subunit beta type-8	P28062	1,312725188	Cytoplasm (Soluble) + Nucleus	3	5
Proteasome subunit beta type-7	Q99436	0,276404965	Cytoplasm (Soluble) + Nucleus	6	3
Proteasome subunit beta type-6	P28072	0,833235524	Cytoplasm (Soluble) + Nucleus	4	3
Proteasome subunit beta type-5	P28074	0,046777528	Cytoplasm (Soluble) + Nucleus	8	4
Proteasome subunit beta type-4	P28070	0,367825214	Cytoplasm (Soluble) + Nucleus	6	1

Proteasome subunit beta type-3	P49720	0,131338242	Cytoplasm (Soluble) + Nucleus	8	4
Proteasome subunit beta type-2	P49721	0,765745521	Cytoplasm (Soluble) + Nucleus	8	2
Proteasome subunit beta type-1	P20618	0,44827517	Cytoplasm (Soluble) + Nucleus	5	1
Proteasome subunit alpha type-7, 8	O14818, Q8TAA3	0,546713635	Cytoplasm (Soluble) + Nucleus	10	9
Proteasome subunit alpha type-6	P60900	0,532336995	Cytoplasm (Soluble) + Nucleus	11	10
Proteasome subunit alpha type-5	P28066	0,588389066	Cytoplasm (Soluble) + Nucleus	9	2
Proteasome subunit alpha type-4	P25789	0,59627778	Cytoplasm (Soluble) + Nucleus	9	12
Proteasome subunit alpha type-3	P25788	0,558439436	Cytoplasm (Soluble) + Nucleus	10	6
Proteasome subunit alpha type-2	P25787	0,058385803	Cytoplasm (Soluble) + Nucleus	9	6
Proteasome subunit alpha type-1	P25786	0,113994325	Cytoplasm (Soluble) + Nucleus	12	4
Proteasome activator complex subunit 1	Q06323	0,084760941	Cytoplasm (Soluble) + Nucleus	14	5
Probable aminopeptidase NPEPL1	Q8NDH3	0,517278353	Cytoplasm (Soluble) + Nucleus	1	6
Prefoldin subunit 3	P61758	0,047021869	Cytoplasm (Soluble) + Nucleus	4	2
NudC domain-containing protein 1	Q96RS6	0,085392215	Cytoplasm (Soluble) + Nucleus	5	4
Nucleoside diphosphate kinase B	P22392	0,012683118	Cytoplasm (Soluble) + Nucleus	14	9
Nucleoside diphosphate kinase A	P15531	0,436501901	Cytoplasm (Soluble) + Nucleus	10	2
Nuclear autoantigenic sperm protein	P49321	0,221156601	Cytoplasm (Soluble) + Nucleus	8	11
Importin-9	Q96P70	0,437441431	Cytoplasm (Soluble) + Nucleus	2	1
Importin-7	O95373	0,282931883	Cytoplasm (Soluble) + Nucleus	5	1
Importin-5	O00410	0,412690269	Cytoplasm (Soluble) + Nucleus	17	17
Importin subunit beta-1	Q14974	0,471395515	Cytoplasm (soluble) + Nucleus	15	7
Importin subunit alpha-3, 4	O00505, O00629	0,206222802	Cytoplasm (Soluble) + Nucleus	4	7
Exportin-2	P55060	0,344299517	Cytoplasm (Soluble) + Nucleus	17	4
Eukaryotic translation initiation factor 4 gamma 1, 3	Q04637, O43432	0,110449943	Cytoplasm (Soluble) + Nucleus	6	29
Eukaryotic translation initiation factor 3 subunit E	P60228	0,022843503	Cytoplasm (Soluble) + Nucleus	7	22
Enolase-phosphatase E1	Q9UHY7	0,37970937	Cytoplasm (Soluble) + Nucleus	2	3
Elongation factor 2	P13639	0,095176194	Cytoplasm (Soluble) + Nucleus	40	1
Elongation factor 1-gamma	P26641	0,081691427	Cytoplasm (Soluble) + Nucleus	15	2
DNA damage-binding protein 1	Q16531	0,193710586	Cytoplasm (Soluble) + Nucleus	20	15
COP9 signalosome complex subunit 1	Q13098	0,096740645	Cytoplasm (Soluble) + Nucleus	6	18
26S proteasome non-ATPase regulatory subunit 11	O00231	0,019720514	Cytoplasm (Soluble) + Nucleus	16	5
14-3-3 protein epsilon	P62258	0,130022923	Cytoplasm (Soluble) + Nucleus	18	7
Transportin-1,2	Q92973, O14787	0,196070642	Cytoplasm (Soluble) + Nucleus	8	12
Transketolase	P29401	0,086360397	Cytoplasm (Soluble) + Nucleus	29	4
Transaldolase	P37837	0,157897021	Cytoplasm (Soluble) + Nucleus	13	5
Thioredoxin reductase 1, cytoplasmic	Q16881	0,094473935	Cytoplasm (Soluble) + Nucleus	16	16
SUMO-activating enzyme subunit 2	Q9UBT2	0,036698974	Cytoplasm (Soluble) + Nucleus	7	6
Stress-induced-phosphoprotein 1	P31948	0,004365458	Cytoplasm (Soluble) + Nucleus	20	6
Ras GTPase-activating protein-binding protein 1	Q13283	0,018574114	Cytoplasm (Soluble) + Nucleus	14	27
Pyruvate kinase PKM, PKL, PKR	P14618, P30613-2, P30613	0,095274147	Cytoplasm (Soluble) + Nucleus	36	9

Small ribosomal subunit protein uS9	P62249	0,024488344	Cytoplasm (Soluble) + Nucleus	7	6
Proteasome activator complex subunit 2	Q9UL46	0,062660644	Cytoplasm (Soluble) + Nucleus	7	4
Nascent polypeptide-associated complex subunit alpha	Q13765	0,010230334	Cytoplasm (Soluble) + Nucleus	4	11
Methionine adenosyltransferase 2 subunit beta	Q9NZL9	0,025789315	Cytoplasm (Soluble) + Nucleus	7	15
Major vault protein	Q14764	0,007084717	Cytoplasm (Soluble) + Nucleus	19	10
Interleukin enhancer-binding factor 2	Q12905	0,06914371	Cytoplasm (Soluble) + Nucleus	11	4
Exportin-1	O14980	0,055068054	Cytoplasm (Soluble) + Nucleus	4	22
Thioredoxin-like protein 1	O43396	0,055890301	Cytoplasm (Soluble) + Nucleus	8	5
Signal transducer and activator of transcription 1-alpha/beta	P42224	0,06667013	Cytoplasm (Soluble) + Nucleus	25	20
COP9 signalosome complex subunit 5	Q92905	0,021713275	Cytoplasm (soluble) + Nucleus + Cytoplasmic vesicle	6	3
COP9 signalosome complex subunit 4	Q9BT78	0,021233716	Cytoplasm (soluble) + Nucleus + Cytoplasmic vesicle	14	6
Importin subunit alpha-1	P52292	0,032590952	Cytoplasm (Soluble) + Nucleus + ER/Golgi	4	7
Eukaryotic translation initiation factor 5A- 1, 2	P63241, Q9GZV4	0,005316955	Cytoplasm (Soluble) + Nucleus + ER/Golgi	8	9
Protein arginine N-methyltransferase 1, 8	Q99873, Q9NR22	0,089407071	Cytoplasm (Soluble) + Nucleus + Cell membrane	10	11
Elongation factor 1-alpha 1, 2	P68104, Q05639	0,020186806	Cytoplasm (Soluble) + Nucleus + Cell membrane	20	15
Serine/threonine-protein phosphatase 2A 65 kDa regulatory subunit A alpha isoform, beta isoform	P30153, P30154-5	0,143527695	Cytoplasm (Soluble) + Nucleus + Cell membrane	14	8
Parkinson disease protein 7	Q99497	0,05008011	Cytoplasm (Soluble) + Nucleus + Cell membrane + ER/Golgi + Mitochondria	11	3
Heat shock protein HSP 90-beta	P08238	0,200883254	Cytoplasm (Soluble) + Nucleus + Cell membrane + Extracellular (secreted)	34	2
Lysine--tRNA ligase	Q15046	0,236225384	Cytoplasm (Soluble) + Nucleus + Cell membrane + Extracellular (secreted) + Mitochondria	12	6
Heat shock cognate 71 kDa protein	P11142	0,10261709	Cytoplasm (Soluble) + Nucleus + Cell membrane + Lysosome	34	15
Heat shock protein HSP 90-alpha, Heat shock protein HSP 90-alpha A2	P07900, Q14568	0,504135476	Cytoplasm (Soluble) + Nucleus + Cell membrane + Mitochondria	42	6
Y-box-binding protein 1, 2, 3	P67809, P16989, Q9Y2T7	0,030567686	Cytoplasm (Soluble) + Nucleus + Cytoplasmic granule + Extracellular (Secreted)	8	8
Adenosylhomocysteinase	P23526	0,215119384	Cytoplasm (Soluble) + Nucleus + ER/Golgi	13	2
Transitional endoplasmic reticulum ATPase	P55072	0,254166948	Cytoplasm (Soluble) + Nucleus + ER/Golgi	41	18
Peptidyl-prolyl cis-trans isomerase A	P62937	0,010193577	Cytoplasm (soluble) + Nucleus + Extracellular (Secreted)	12	16
Heat shock 70 kDa protein 6;Putative heat shock 70 kDa protein 7	P17066, P48741	0,095558618	Cytoplasm (solube) + Nucleus + Extracellular (Secreted)	9	2

Protein-glutamine gamma-glutamyltransferase 2, 5	P21980, O43548	0,004491582	Cytoplasm (Soluble) + Nucleus + Extracellular (secreted) + Cell membrane + Mitochondria	22	7
Ubiquitin carboxyl-terminal hydrolase 5, 13	P45974, Q92995	0,025137463	Cytoplasm (Soluble) + Nucleus + Lysosome	10	8
Polyubiquitin-B, C, Ubiquitin-ribosomal protein; eL40 fusion protein, eS31 fusion protein	P0CG48, P62987, P62979, P0CG47	0,053713222	Cytoplasm (Soluble) + Nucleus + Mitochondria	9	21
Glutathione S-transferase P	P09211	0,08084837	Cytoplasm (Soluble) + Nucleus + Mitochondria	13	5
Ubiquitin-like modifier-activating enzyme 1	P22314	0,106096376	Cytoplasm (Soluble) + Nucleus + Mitochondria	26	8
Polyubiquitin-B, C, Ubiquitin-ribosomal protein: eL40 fusion protein, eS31 fusion protein	P0CG48, P62987, P62979, P0CG47	0,053713222	Cytoplasm (Soluble) + Nucleus + Mitochondria	9	21
Superoxide dismutase [Cu-Zn]	P00441	0,132264916	Cytoplasm (Soluble) + Nucleus + Mitochondria	5	2
Clathrin heavy chain 1, 2	Q00610, P53675	0,178523896	Cytoplasmic vesicle + Cell membrane + Cytoplasm (Cytoskeleton/Motor)	38	19
Protein transport protein Sec24C	P53992	0,330533111	Cytoplasmic vesicle + Cytoplasm (soluble) + ER/Golgi	2	2
Guanylate-binding protein 1	P32455	0,572926844	Cytoplasmic vesicle + Cytoplasm (Soluble) + ER/Golgi + Cell membrane + Extracellular (Secreted)	2	4
SEC23-interacting protein	Q9Y6Y8	3,978171193	Cytoplasmic vesicle + ER/Golgi	3	2
Carboxypeptidase E	P16870	Only in EVs	Cytoplasmic vesicle + Extracellular (secreted)	1	1
Protein TFG	Q92734	0,384239266	ER/Golgi	7	16
Protein disulfide-isomerase A4	P13667	0,051851558	ER/Golgi	29	3
Protein disulfide-isomerase A3	P30101	0,00171134	ER/Golgi	30	17
Procollagen-lysine,2-oxoglutarate 5-dioxygenase 2	O00469	0,443000754	ER/Golgi	23	6
Procollagen-lysine,2-oxoglutarate 5-dioxygenase 1	Q02809	1,701069122	ER/Golgi	13	4
Polypeptide N-acetylgalactosaminyltransferase 6	Q8NCL4	Only in EVs	ER/Golgi	1	6
Peptidyl-prolyl cis-trans isomerase FKBP10	Q96AY3	0,026070611	ER/Golgi	8	6
Neutral alpha-glucosidase AB	Q14697	0,014890669	ER/Golgi	32	5
Heat shock 70 kDa protein 13	P48723	2,922616933	ER/Golgi	4	1
Golgi membrane protein 1	Q8NBJ4	Only in EVs	ER/Golgi	1	3
Glucosidase 2 subunit beta	P14314	0,029919244	ER/Golgi	17	9
Exostosin-1	Q16394	Only in EVs	ER/Golgi	1	2
Endoplasmic reticulum aminopeptidase 1	Q9NZ08	1,088119511	ER/Golgi	9	2
Beta-1,4-galactosyltransferase 5	O43286	17,45886792	ER/Golgi	1	1
Beta-1,3-galactosyl-O-glycosyl-glycoprotein beta-1,6-N-acetylglucosaminyltransferase	Q02742	Only in EVs	ER/Golgi	15	1
Alpha-mannosidase 2	Q16706	Only in EVs	ER/Golgi	1	1
Vesicular integral-membrane protein VIP36	Q12907	Only in EVs	ER/Golgi	5	6
Transmembrane protein 132A	Q24JP5	Only in EVs	ER/Golgi	10	7
Peptidyl-prolyl cis-trans isomerase FKBP9	O95302	0,198641518	ER/Golgi	3	5
Endoplasmin	P14625	0,053457711	ER/Golgi	40	14

Endoplasmic reticulum aminopeptidase 2	Q6P179	6,984576514	ER/Golgi	3	6
Endoplasmic reticulum chaperone BiP	P11021	0,060561827	ER/Golgi + Extracellular (Secreted) +Cytoplasm (Soluble)	36	3
Protein disulfide-isomerase	P07237	0,079611849	ER/Golgi + Cell membrane	30	32
Calsyntenin-3	Q9BQT9	Only in EVs	ER/Golgi + Cell membrane	1	1
45 kDa calcium-binding protein	Q9BRK5	Only in EVs	ER/Golgi + Cell membrane + Cytoplasm (soluble)	1	1
Ribosome-binding protein 1, Ninein	Q9P2E9, Q8N4C6-6	0,089042913	ER/Golgi + Cytoplasm (Soluble)	8	7
Nucleobindin-1	Q02818	2,543948836	ER/Golgi + Cytoplasm (Soluble) + Extracellular (Secreted)	18	8
Calreticulin	P27797	0,145048388	ER/Golgi + Cytoplasm (Soluble) + Extracellular (Secreted) + Cytoplasmic vesicle	14	4
AP-1 complex subunit beta-1	Q10567	0,13629334	ER/Golgi + Cytoplasmic vesicle	6	5
AP-1 complex subunit gamma-1	O43747	0,514985812	ER/Golgi + Cytoplasmic vesicle + Cell membrane + Cytoplasm (Soluble)	1	2
Ras-related protein Rab-6A, 6B, 39A	P20340, Q9NRW1, Q14964	0,133555591	ER/Golgi + Cytoplasmic vesicle + Cell membrane + Lysosome	2	10
Cation-independent mannose-6-phosphate receptor	P11717	Only in EVs	ER/Golgi + Endosome	1	2
N-acetylglucosamine-1-phosphotransferase subunit gamma	Q9UJJ9	Only in EVs	ER/Golgi + Extracellular (Secreted)	5	5
Exostosin-like 2;Processed exostosin-like 2	Q9UBQ6	7,238031776	ER/Golgi + Extracellular (Secreted)	2	4
Calumenin	O43852	0,009820575	ER/Golgi + Extracellular (Secreted)	14	16
Alpha-1-antitrypsin, Alpha-1-antitrypsin-related protein	P01009, P20848	Only in EVs	ER/Golgi + Extracellular (Secreted)	5	1
Procollagen-lysine,2-oxoglutarate 5-dioxygenase 3	O60568	0,078058605	ER/Golgi + Extracellular (secreted)	7	4
Extracellular serine/threonine protein kinase FAM20C, Pseudokinase FAM20A	Q8IXL6, Q96MK3	Only in EVs	ER/Golgi + Extracellular (secreted)	3	2
Exostosin-2	Q93063	Only in EVs	ER/Golgi + Extracellular (secreted)	2	2
Xylosyltransferase 2	Q9H1B5	Only in EVs	ER/Golgi + Extracellular (secreted)	3	5
Arylsulfatase A	P15289	Only in EVs	ER/Golgi + Lysosome	9	1
Calnexin	P27824	0,029134153	ER/Golgi + Mitochondria	9	20
Protein CREG1	O75629	Only in EVs	Extracellular (Secreted)	9	6
Probable serine carboxypeptidase CPVL	Q9H3G5	Only in EVs	Extracellular (Secreted)	2	6
Plasminogen activator inhibitor 1	P05121	5,620889961	Extracellular (Secreted)	19	2
Plasma protease C1 inhibitor	P05155	Only in EVs	Extracellular (Secreted)	5	6
Plasma alpha-L-fucosidase	Q9BTY2	Only in EVs	Extracellular (Secreted)	18	6
Pentraxin-related protein PTX3	P26022	Only in EVs	Extracellular (Secreted)	3	5
Nidogen-1	P14543	Only in EVs	Extracellular (Secreted)	1	5
Midkine	P21741	Only in EVs	Extracellular (Secreted)	7	5
Metalloproteinase inhibitor 2	P16035	Only in EVs	Extracellular (Secreted)	1	5
Metalloproteinase inhibitor 1	P01033	Only in EVs	Extracellular (Secreted)	1	5
Matrix metalloproteinase-9 (MMP-9)	P14780	Only in EVs	Extracellular (Secreted)	1	4
Lumican	P51884	Only in EVs	Extracellular (Secreted)	2	4
Latent-transforming growth factor beta-binding protein 2	Q14767	Only in EVs	Extracellular (Secreted)	6	4

Laminin subunit gamma-1, 3	P11047, Q9Y6N6	4,194122616	Extracellular (Secreted)	26	2
Laminin subunit beta-1	P07942	9,773043075	Extracellular (Secreted)	26	15
Laminin subunit alpha-5	O15230	Only in EVs	Extracellular (Secreted)	4	4
Laminin subunit alpha-4	Q16363	Only in EVs	Extracellular (Secreted)	3	4
Interleukin-6	P05231	Only in EVs	Extracellular (Secreted)	4	4
Inter-alpha-trypsin inhibitor heavy chain H4	Q14624	Only in EVs	Extracellular (Secreted)	12	4
Insulin-like growth factor-binding protein 7	Q16270	Only in EVs	Extracellular (Secreted)	2	3
Insulin-like growth factor-binding protein 6	P24592	Only in EVs	Extracellular (Secreted)	5	3
Inhibin beta A chain	P08476	Only in EVs	Extracellular (Secreted)	4	3
Immunoglobulin lambda-like polypeptide 5	B9A064	Only in EVs	Extracellular (Secreted)	14	3
Hemopexin	P02790	Only in EVs	Extracellular (Secreted)	4	3
Growth arrest-specific protein 6	Q14393	Only in EVs	Extracellular (Secreted)	8	3
Gremlin-1;Gremlin-2	O60565, Q9H772	Only in EVs	Extracellular (Secreted)	2	3
Granulocyte colony-stimulating factor	P09919	Only in EVs	Extracellular (Secreted)	1	3
Galectin-3-binding protein	Q08380	106,4886284	Extracellular (Secreted)	28	10
Follistatin-related protein 1	Q12841	99,28657667	Extracellular (Secreted)	9	4
Fibulin-2	P98095	Only in EVs	Extracellular (Secreted)	2	3
Fibulin-1	P23142	Only in EVs	Extracellular (Secreted)	13	3
Fibulin-1	P23142	Only in EVs	Extracellular (Secreted)	3	3
Fibronectin	P02751	264,1302497	Extracellular (Secreted)	64	19
Fibromodulin	Q06828	Only in EVs	Extracellular (secreted)	2	3
Fibrillin-1	P35555	Only in EVs	Extracellular (Secreted)	1	3
Extracellular matrix protein 1	Q16610	Only in EVs	Extracellular (Secreted)	2	2
Epididymis-specific alpha-mannosidase	Q9Y2E5	Only in EVs	Extracellular (Secreted)	3	2
EGF-containing fibulin-like extracellular matrix protein 1	Q12805	Only in EVs	Extracellular (Secreted)	1	2
Dickkopf-related protein 3	Q9UBP4	Only in EVs	Extracellular (Secreted)	2	2
Decorin	P07585	Only in EVs	Extracellular (Secreted)	1	2
Cystatin-C	P01034	Only in EVs	Extracellular (Secreted)	3	2
Complement factor I	P05156	Only in EVs	Extracellular (Secreted)	5	2
Complement factor H, Complement factor H-related protein 3	P08603, Q02985	Only in EVs	Extracellular (Secreted)	1	2
Complement factor B	P00751	Only in EVs	Extracellular (Secreted)	2	2
Complement C4-A, B	P0C0L4, P0C0L5	Only in EVs	Extracellular (Secreted)	5	2
Complement C3	P01024	993,0722234	Extracellular (Secreted)	102	13
Complement C1s subcomponent, Mannan-binding lectin serine protease 1	P09871, P48740	Only in EVs	Extracellular (Secreted)	13	2
Complement C1r subcomponent	P00736	Only in EVs	Extracellular (Secreted)	3	2
Complement C1r subcomponent-like protein	Q9NZP8	Only in EVs	Extracellular (Secreted)	4	2
Collagen and calcium-binding EGF domain-containing protein 1	Q6UXH8	Only in EVs	Extracellular (Secreted)	2	2
Collagen alpha-2(VI) chain	P12110	Only in EVs	Extracellular (secreted)	1	2
Collagen alpha-2(V) chain	P05997	Only in EVs	Extracellular (Secreted)	2	2
Collagen alpha-2(I) chain	P08123	Only in EVs	Extracellular (Secreted)	3	2

Collagen alpha-1(VI) chain	P12109	Only in EVs	Extracellular (Secreted)	1	2
Collagen alpha-1(I) chain	P02452	Only in EVs	Extracellular (Secreted)	5	2
Coiled-coil domain-containing protein 80	Q76M96	Only in EVs	Extracellular (Secreted)	5	2
Carboxypeptidase A4	Q9UI42	Only in EVs	Extracellular (Secreted)	2	1
Biotinidase	P43251	Only in EVs	Extracellular (Secreted)	1	1
Biglycan	P21810	Only in EVs	Extracellular (Secreted)	1	1
Basement membrane-specific heparan sulfate proteoglycan core protein	P98160	Only in EVs	Extracellular (Secreted)	2	1
Annexin A2	P07355	0,0107592	Extracellular (Secreted)	31	25
Angiopoietin-related protein 4	Q9BY76	Only in EVs	Extracellular (Secreted)	1	1
Xaa-Pro dipeptidase	P12955	0,758346802	Extracellular (Secreted)	4	17
Versican core protein	P13611	Only in EVs	Extracellular (Secreted)	18	8
Tumor necrosis factor-inducible gene 6 protein	P98066	Only in EVs	Extracellular (Secreted)	3	1
Tubulointerstitial nephritis antigen-like	Q9GZM7	Only in EVs	Extracellular (Secreted)	1	3
Transforming growth factor-beta-induced protein ig-h3	Q15582	67,0531422	Extracellular (Secreted)	38	8
Transforming growth factor beta-2 proprotein	P61812	Only in EVs	Extracellular (Secreted)	3	2
Transforming growth factor beta-1 proprotein	P01137	Only in EVs	Extracellular (Secreted)	3	4
Thrombospondin-1, 2, 3	P07996, P35442, P49746	1874,114703	Extracellular (Secreted)	45	13
Testican-1	Q08629	Only in EVs	Extracellular (Secreted)	4	2
Sushi repeat-containing protein SRPX2	O60687	Only in EVs	Extracellular (secreted)	5	4
Sushi repeat-containing protein SRPX	P78539	Only in EVs	Extracellular (Secreted)	2	5
Stanniocalcin-2	O76061	Only in EVs	Extracellular (Secreted)	4	3
Stanniocalcin-1	P52823	Only in EVs	Extracellular (Secreted)	1	1
SPARC	P09486	59,40790028	Extracellular (Secreted)	11	3
Serotransferrin	P02787	859,6107647	Extracellular (Secreted)	84	7
Semaphorin-3F	Q13275	Only in EVs	Extracellular (Secreted)	1	3
Semaphorin-3C	Q99985	Only in EVs	Extracellular (Secreted)	2	1
Retinol-binding protein 4	P02753	Only in EVs	Extracellular (Secreted)	1	2
Beta-2-microglobulin	P61769	0,204977826	Extracellular (Secreted)	4	3
Aminopeptidase B	Q9H4A4	0,2269684	Extracellular (Secreted)	3	5
Immunoglobulin kappa constant	P01834	Only in EVs	Extracellular (Secreted) + Cell membrane	3	3
Immunoglobulin heavy constant alpha 1, 2	P01876, P01877	Only in EVs	Extracellular (Secreted) + Cell membrane	25	3
Immunoglobulin heavy constant gamma 3	P01860	Only in EVs	Extracellular (Secreted) + Cell membrane	1	3
Immunoglobulin heavy constant gamma 2	P01859	Only in EVs	Extracellular (Secreted) + Cell membrane	1	3
Immunoglobulin heavy constant gamma 1	P01857	43,22039612	Extracellular (Secreted) + Cell membrane	10	3
Agrin	O00468, O00468-2	Only in EVs	Extracellular (Secreted) + Cell membrane	2	1
Vasorin	Q6EMK4	Only in EVs	Extracellular (Secreted) + Cell membrane	5	1
Tissue factor (CD142)	P13726	Only in EVs	Extracellular (Secreted) + Cell membrane	1	2
Retinoic acid receptor responder protein 1	P49788	Only in EVs	Extracellular (Secreted) + Cell membrane	5	2
Hepatocyte growth factor receptor	P08581	Only in EVs	Extracellular (secreted) + Cell membrane	1	3

Dystroglycan 1	Q14118	Only in EVs	Extracellular (Secreted) + Cell membrane + Cytoplasm (Cytoskeleton/Motor) + Nucleus	3	2
Protein S100-A10	P60903	0,009476913	Extracellular (secreted) + Cell membrane + Cytoplasm (Soluble) + Nucleus	6	1
Macrophage migration inhibitory factor	P14174	0,004844458	Extracellular (Secreted) + Cytoplasm (soluble)	5	1
Galectin-1	P09382	0,025477011	Extracellular (Secreted) + Cytoplasm (Soluble)	11	2
C-type lectin domain family 11 member A	Q9Y240	Only in EVs	Extracellular (Secreted) + Cytoplasm (soluble)	4	1
Adipocyte enhancer-binding protein 1	Q8IUX7	Only in EVs	Extracellular (Secreted) + Cytoplasm (soluble) + Nucleus	3	1
Serine protease HTRA1	Q92743	Only in EVs	Extracellular (Secreted) + Cytoplasm (soluble) + Cell membrane	2	3
Neuroserpin	Q99574	Only in EVs	Extracellular (Secreted) + Cytoplasmic vesicle	2	5
Peroxidasin homolog	Q92626	Only in EVs	Extracellular (Secreted) + ER/Golgi	1	6
Bone morphogenetic protein 1	P13497	Only in EVs	Extracellular (Secreted) + ER/Golgi	1	1
Sulfhydryl oxidase 1	O00391	Only in EVs	Extracellular (Secreted) + ER/Golgi	13	3
Serglycin	P10124	Only in EVs	Extracellular (Secreted) + ER/Golgi + Cytoplasmic granules	4	1
Prosaposin	P07602	17,84942606	Extracellular (Secreted) + Lysosome	18	4
Progranulin	P28799	Only in EVs	Extracellular (Secreted) + Lysosome	5	6
Gamma-interferon-inducible lysosomal thiol reductase	P13284	Only in EVs	Extracellular (Secreted) + Lysosome	6	3
Gamma-glutamyl hydrolase	Q92820	27,10721723	Extracellular (Secreted) + Lysosome	9	4
Phospholipase A2 group XV	Q8NCC3	Only in EVs	Extracellular (Secreted) + Lysosome + Cell membrane	5	6
72 kDa type IV collagenase (MMP-2)	P08253	Only in EVs	Extracellular (Secreted) + Nucleus + Cytoplasm (soluble) + Mitochondia	5	1
Putative phospholipase B-like 2	Q8NHP8	Only in EVs	Lysosome	18	7
Peroxiredoxin-6	P30041	0,021013608	Lysosome	19	1
N-sulphoglucosamine sulphohydrolase	P51688	Only in EVs	Lysosome	2	5
N-acetylglucosamine-6-sulfatase	P15586	0,332585157	Lysosome	6	6
Lysosome membrane protein 2	Q14108	Only in EVs	Lysosome	10	4
Lysosomal Pro-X carboxypeptidase	P42785	1,287029865	Lysosome	4	13
Lysosomal alpha-mannosidase	O00754	Only in EVs	Lysosome	1	4
Lysosomal acid lipase/cholesteryl ester hydrolase	P38571	Only in EVs	Lysosome	22	4
Lysosomal acid glucosylceramidase	P04062	0,847836622	Lysosome	4	8
Legumain	Q99538	Only in EVs	Lysosome	1	4
Iduronate 2-sulfatase	P22304	Only in EVs	Lysosome	2	3
Dipeptidyl peptidase 1	P53634	1,934008494	Lysosome	5	16
Cathepsin Z	Q9UBR2	1,071750742	Lysosome	5	18
Cathepsin B	P07858	1,933665949	Lysosome	12	20
Beta-hexosaminidase subunit alpha	P06865	11,90052351	Lysosome	10	13
Alpha-galactosidase A	P06280	4,103112111	Lysosome	2	6
Tripeptidyl-peptidase 1	O14773	4,409763955	Lysosome	7	10

Tissue alpha-L-fucosidase	P04066	6,239264351	Lysosome	5	1
Lysosomal protective protein	P10619	0,222467283	Lysosome	4	8
Beta-mannosidase	O00462	Only in EVs	Lysosome	1	1
Cathepsin D	P07339	8,903166735	Lysosome + Extracellular	14	13
Beta-galactosidase	P16278	0,801009421	Lysosome + Cytoplasm (Soluble)	9	9
Beta-hexosaminidase subunit beta	P07686	2,948584633	Lysosome + Cytoplasmic vesicles	8	4
Procathepsin L1	P07711	Only in EVs	Lysosome + Cytoplasmic vesicles + Cell membrane + Extracellular (secreted)	13	6
Superoxide dismutase [Mn], mitochondrial	P04179	0,066830607	Mitochondria	6	10
Nardilysin	O43847	0,511304822	Mitochondria	3	4
Glutathione reductase, mitochondrial	P00390	0,277510662	Mitochondria	3	7
Enoyl-CoA delta isomerase 1, mitochondrial	P42126	0,015625084	Mitochondria	5	4
Citrate synthase, mitochondrial	O75390	0,024595825	Mitochondria	8	19
10 kDa heat shock protein, mitochondrial	P61604	0,003748935	Mitochondria	11	4
Single-stranded DNA-binding protein, mitochondrial	Q04837	0,014343374	Mitochondria	9	4
Malate dehydrogenase, mitochondrial	P40926	0,004463727	Mitochondria	14	3
60 kDa heat shock protein, mitochondrial	P10809	0,012089292	Mitochondria	46	15
Thioredoxin-dependent peroxide reductase, mitochondrial	P30048	0,029171586	Mitochondria + Cytoplasm (soluble) + Endosome	5	2
Fumarate hydratase, mitochondrial	P07954	0,127507492	Mitochondria + Cytoplasm (soluble) + Nucleus	8	4
NAD(P)H-hydrate epimerase	Q8NCW5	0,190243561	Mitochondria + Extracellular (secreted)	4	7
Prothymosin alpha	P06454	0,195032119	Nucleus	5	6
Protein FAM53C	Q9NYF3	Only in EVs	Nucleus	11	7
Lupus La protein	P05455	0,026499055	Nucleus	11	4
Histone H4	P62805	0,011203444	Nucleus	13	1
Histone H3.1, H3.1t, H3.2, H3.3, H3.3C	Q71DI3, Q16695, P84243, P68431, Q6NXT2	0,006711794	Nucleus	5	11
Histone H2B type 1-A, C/E/F/G/I, D, H, K, L, M, N, Histone H2B type F-S, Histone H2B type 2-F, K1	P62807, P57053, O60814, Q99879, Q99877, Q93079, Q5QNW6, P58876, Q99880, Q96A08	0,009061098	Nucleus	10	13
Heterogeneous nuclear ribonucleoproteins C1/C2, C-like 1, RNA-binding Raly-like protein	P07910, O60812, Q86SE5	0,019317834	Nucleus	14	29
Elongation factor 1-delta	P29692	0,013910303	Nucleus	12	24
DNA-directed RNA polymerase II subunit RPB2	P30876	19,00842836	Nucleus	2	3
Chromobox protein homolog 3	Q13185	0,066564328	Nucleus	3	4
X-ray repair cross-complementing protein 6	P12956	0,024563183	Nucleus	14	3
X-ray repair cross-complementing protein 5	P13010	0,08918189	Nucleus	11	1

Ubiquitin-conjugating enzyme E2 variant 1	Q13404	0,009122788	Nucleus	7	11
Splicing factor U2AF 65 kDa subunit	P26368	0,080966836	Nucleus	4	2
Splicing factor 3B subunit 3	Q15393	0,013569561	Nucleus	15	5
Ras-responsive element-binding protein 1	Q92766	Only in EVs	Nucleus	1	6
Histone-binding protein RBBP4 and RBBP7	Q09028, Q16576	0,119362283	Nucleus	8	19
Histone H2A J, V, X, type 1, type 1 A, D, H, J, type 2 A, C	Q99878, Q96KK5, Q9BTM1, Q16777, Q6FI13, P20671, P0C0S8, Q96QV6, P16104, Q71UI9-5	0,009356359	Nucleus	7	15
ADP-sugar pyrophosphatase	Q9UKK9	0,118004674	Nucleus	8	6
Nesprin-2	Q8WXH0	Only in EVs	Nucleus + Cell membrane + Cytoplasm (Cytoskeleton/motor) + Mitochondia	1	5
Chloride intracellular channel protein 1	O00299	0,049789215	Nucleus + Cell membrane + Cytoplasm (Soluble) + ER/Golgi	13	1
Nucleophosmin	P06748	0,023168295	Nucleus + Cytoplasm (Cytoskeleton/Motor)	17	14
BRCA2 and CDKN1A-interacting protein	Q9P287	0,06749811	Nucleus + Cytoplasm (Cytoskeleton/Motor)	4	4
Cell cycle and apoptosis regulator protein 2	Q8N163	0,123842461	Nucleus + Cytoplasm (Soluble and Cytoskeleton/Motor)	3	5
ATP-dependent RNA helicase A	Q08211	0,01372956	Nucleus + Cytoplasm (Soluble and Cytoskeleton/Motor)	11	2
Nucleosome assembly protein 1-like 4	Q99733	0,037209162	Nucleus + Cytoplasm (Soluble)	7	14
Macrophage-capping protein	P40121	0,009374569	Nucleus + Cytoplasm (Soluble)	13	5
Heterogeneous nuclear ribonucleoprotein D0	Q14103	0,008295045	Nucleus + Cytoplasm (Soluble)	12	10
Heterogeneous nuclear ribonucleoprotein A1	P09651	0,004585109	Nucleus + Cytoplasm (Soluble)	15	45
Heterogeneous nuclear ribonucleoprotein A/B	Q99729	0,021741617	Nucleus + Cytoplasm (Soluble)	10	7
Cullin-4 A, B	Q13620, Q13619	0,049870456	Nucleus + Cytoplasm (Soluble)	9	23
Acidic leucine-rich nuclear phosphoprotein 32 family member B	Q92688	0,20975889	Nucleus + Cytoplasm (Soluble)	5	2
Protein S100-A6	P06703	0,11200034	Nucleus + Cytoplasm (Soluble)	6	2
Proteasome activator complex subunit 3	P61289	0,051070957	Nucleus + Cytoplasm (Soluble)	4	8
Poly(rC)-binding protein 1	Q15365	0,008787391	Nucleus + Cytoplasm (Soluble)	8	2
Nucleosome assembly protein 1-like 1	P55209	0,101137738	Nucleus + Cytoplasm (Soluble)	5	18
Nucleolin	P19338	0,095118799	Nucleus + Cytoplasm (Soluble)	19	11
Nuclear transport factor 2	P61970	0,122598439	Nucleus + Cytoplasm (Soluble)	3	5
Heterogeneous nuclear ribonucleoprotein K	P61978	0,00539973	Nucleus + Cytoplasm (Soluble)	21	6
GTP-binding nuclear protein Ran	P62826	0,02953734	Nucleus + Cytoplasm (Soluble)	11	7
Alpha-ketoglutarate-dependent dioxygenase FTO	Q9C0B1	6,065310944	Nucleus + Cytoplasm (Soluble)	2	8
Testis-specific Y-encoded-like protein 2	Q9H2G4	Only in EVs	Nucleus + Cytoplasm (Soluble)	1	1
Spliceosome RNA helicase DDX39B	Q13838	0,081901352	Nucleus + Cytoplasm (Soluble)	15	23

Heterogeneous nuclear ribonucleoproteins A2/B1	P22626	0,001426718	Nucleus + Cytoplasm (Soluble) + Cytoplasmic granule + Extracellular secreted	17	12
Heterogeneous nuclear ribonucleoprotein R	O43390	0,018693575	Nucleus + Cytoplasm (Soluble) + ER/Golgi	9	9
Heterogeneous nuclear ribonucleoprotein Q	O60506	0,009133092	Nucleus + Cytoplasm (Soluble) + ER/Golgi	16	16
Acidic leucine-rich nuclear phosphoprotein 32 family member A	P39687	0,246978368	Nucleus + Cytoplasm (Soluble) + ER/Golgi	8	5
Thioredoxin	P10599	0,034423005	Nucleus + Cytoplasm (Soluble) + Extracellular (Secreted)	8	2
Phospholipid transfer protein	P55058	Only in EVs	Nucleus + Extracellular (Secreted)	11	6
Nicotinamide phosphoribosyltransferase	P43490	0,056794935	Nucleus + Extracellular (Secreted) + Cytoplasm (Soluble)	20	26
Lysyl oxidase homolog 2	Q9Y4K0	Only in EVs	Nucleus + Extracellular (secreted) + ER/Golgi	1	4
Histone acetyltransferase type B catalytic subunit	O14929	0,287592931	Nucleus + Mitochondria	3	2
Protein PBDC1	Q9BVG4	0,062656557	Unknown	2	1
Putative uncharacterized protein C5orf58	C9J3I9	Only in EVs	Unknown	1	3
